# The relationship between viral clearance rates and disease progression in early symptomatic COVID-19: a systematic review and meta-regression analysis

**DOI:** 10.1093/jac/dkae045

**Published:** 2024-02-22

**Authors:** Shivani Singh, Simon Boyd, William H K Schilling, James A Watson, Mavuto Mukaka, Nicholas J White

**Affiliations:** Faculty of Tropical Medicine, Mahidol Oxford Tropical Medicine Research Unit, Mahidol University, Bangkok, Thailand; Faculty of Tropical Medicine, Mahidol Oxford Tropical Medicine Research Unit, Mahidol University, Bangkok, Thailand; Nuffield Department of Medicine, Centre for Tropical Medicine and Global Health, Oxford University, Oxford, UK; Faculty of Tropical Medicine, Mahidol Oxford Tropical Medicine Research Unit, Mahidol University, Bangkok, Thailand; Nuffield Department of Medicine, Centre for Tropical Medicine and Global Health, Oxford University, Oxford, UK; Nuffield Department of Medicine, Centre for Tropical Medicine and Global Health, Oxford University, Oxford, UK; Biostatistics Department, Oxford University Clinical Research Unit, 764 Vo Van Kiet, Quan 5, Ho Chi Minh City, Vietnam; Faculty of Tropical Medicine, Mahidol Oxford Tropical Medicine Research Unit, Mahidol University, Bangkok, Thailand; Nuffield Department of Medicine, Centre for Tropical Medicine and Global Health, Oxford University, Oxford, UK; Faculty of Tropical Medicine, Mahidol Oxford Tropical Medicine Research Unit, Mahidol University, Bangkok, Thailand; Nuffield Department of Medicine, Centre for Tropical Medicine and Global Health, Oxford University, Oxford, UK

## Abstract

**Background:**

Effective antiviral drugs accelerate viral clearance in acute COVID-19 infections; the relationship between accelerating viral clearance and reducing severe clinical outcomes is unclear.

**Methods:**

A systematic review was conducted of randomized controlled trials (RCTs) of antiviral therapies in early symptomatic COVID-19, where viral clearance data were available. Treatment benefit was defined clinically as the relative risk of hospitalization/death during follow-up (≥14 days), and virologically as the SARS-CoV-2 viral clearance rate ratio (VCRR). The VCRR is the ratio of viral clearance rates between the intervention and control arms. The relationship between the clinical and virological treatment effects was assessed by mixed-effects meta-regression.

**Results:**

From 57 potentially eligible RCTs, VCRRs were derived for 44 (52 384 participants); 32 had ≥1 clinical endpoint in each arm. Overall, 9.7% (R^2^) of the variation in clinical benefit was explained by variation in VCRRs with an estimated linear coefficient of −0.92 (95% CI: −1.99 to 0.13; *P* = 0.08). However, this estimate was highly sensitive to the inclusion of the recent very large PANORAMIC trial. Omitting this outlier, half the variation in clinical benefit (R^2^ = 50.4%) was explained by variation in VCRRs [slope −1.47 (95% CI −2.43 to −0.51); *P* = 0.003], i.e. higher VCRRs were associated with an increased clinical benefit.

**Conclusion:**

Methods of determining viral clearance in COVID-19 studies and the relationship to clinical outcomes vary greatly. As prohibitively large sample sizes are now required to show clinical treatment benefit in antiviral therapeutic assessments, viral clearance is a reasonable surrogate endpoint.

## Introduction

COVID-19 is characterized by rapid viral replication early in the acute infection. Progression to severe disease occurs in a minority of patients after an interval of approximately 1 week. Whereas the initial symptoms result directly from the SARS-CoV-2 burden, the later severe phase with potentially lethal pneumonitis results from immunopathogenesis.^1^ Antiviral therapies are, therefore, expected to provide greatest therapeutic benefit early in the course of infection (i.e. within the first week) when viral densities are highest. Several antiviral interventions have been shown to reduce subsequent all-cause hospitalization and death.^2–5^ However, as the pandemic has evolved the incidence of these severe clinical endpoints has declined substantially. Among COVID-19 cases referred to hospital, inflammatory viral pneumonitis, the main pathological process leading to death, is proportionally less. This now limits the ability of antiviral trials in COVID-19 to detect a treatment effect if prevention of all-cause hospitalization or death is their primary endpoint. The rate of viral clearance from the nasopharynx or oropharynx has been proposed as a surrogate endpoint in COVID-19,^6,7^ but the relationship between acceleration in viral clearance and prevention of disease progression is unclear.

In general, in infectious diseases, pathogen clearance correlates with clinical responses.^8^ Viral load reductions are established as surrogate endpoints of therapeutic efficacy in several different chronic viral infections, including HIV, Hepatitis B and C, and CMV infections in organ transplant patients.^9–12^ This is not the case for acute viral infections. Outpatient trials of antivirals and neutralizing monoclonal antibodies in COVID-19 (most of which were conducted early in the pandemic in unvaccinated populations) demonstrated both strong evidence of clinical benefit and substantial acceleration in viral clearance.^2,3^ Several studies have shown an association between slow viral clearance and increased risk of severe outcomes.^13,14^ High viral loads in hospitalized patients predict mortality.^15^ In a meta-regression analysis of 16 antiviral drug and neutralizing monoclonal antibody trials in COVID-19-infected outpatients,^7^ the relative risk of hospitalization or death was predicted by the magnitude of nasopharyngeal viral load reduction between baseline (Day 0) and Days 5–7. Taken together, these studies provide support for viral load reduction as a surrogate for clinical benefit. This is important because the risk of severe clinical outcomes is now so low that clinical trials of novel antiviral therapies will need to rely surrogate outcomes in order to characterize and compare antiviral efficacy.

Buyse *et al*.^16^ proposed a meta-analytic (meta-regression) approach for validation of surrogate outcomes, which quantifies the association between the treatment effects on the surrogate endpoint (in this case viral clearance rate) and on the clinical endpoint, and quantifies the precision of the prediction with the coefficient of determination (R^2^). Using this approach, we provide an updated evaluation of the relationship between acceleration of SARS-CoV-2 clearance and risk of disease progression (hospitalization or death) in early symptomatic COVID-19.

## Methods

### Overview

The protocol for this systematic review and meta-regression analysis follows the Preferred Reporting Items for Systematic Review and Meta-Analyses (PRISMA) guidelines.^17^ The protocol was registered prospectively with PROSPERO and amended to clarify the statistical analysis plan (CRD42023413208). We summarized the virological and clinical metadata from all Phase 2 and 3 randomized controlled trials (RCTs) that evaluated the use of novel and repurposed antivirals, neutralizing monoclonal antibodies (nMabs), convalescent plasma or interferons in patients with early symptomatic, but uncomplicated, SARS-CoV-2 infections. We included studies of outpatients who had less than 8 days of symptoms (i.e. earlier in the disease course, and before the onset of severe symptoms) or those who were hospitalized for precautionary or pragmatic reasons within 8 days of symptom onset (i.e. also without severe disease).

### Eligibility criteria

Eligible for inclusion were Phase 2 or 3 therapeutic RCTs that included outpatients (or those hospitalized for pragmatic reasons, or to carry out study procedures) with confirmed COVID-19, who presented within 8 days of symptom onset, and that measured SARS-CoV-2 viral loads at baseline and at least one additional timepoint in the first week following enrolment. Viral load in the pharyngeal swab eluate was defined as either a viral genome density value in copies/mL or a cycle threshold (Ct) value. Trials of small-molecule antiviral drugs, nMabs, convalescent plasma, peginterferon lambda and repurposed therapies were all included. We excluded case reports, case series, retrospective studies, non-randomized clinical trials, Phase 1 RCTs, review articles and meta-analyses. Also excluded were RCTs of patients hospitalized for treatment of severe COVID-19 infection, and trials of therapies with predominantly anti-inflammatory mechanisms of action or herbal/non-pharmaceutically standardized therapies.

Full details of the search strategy, methods of data extraction and reporting of viral loads are provided in the Supplementary methods (sections 1–3, available as Supplementary data at *JAC* Online).

### Outcomes

The *primary clinical outcome* analysed was the proportion of patients hospitalized or dead during follow-up (minimum 14 days post-randomization) in the ITT populations. All-cause hospitalization or death was used if available, and COVID-19-related hospitalization or all-cause death was used if the former was not available. The clinical treatment effect was measured as the risk ratio between the intervention and control groups.

The *primary virological outcome* analysed was the estimated median (or mean) rate of pharyngeal viral clearance, expressed as a slope coefficient of the log_10_ RNA copies/mL (quantified in the viral swab eluates) over time. This assumes a first-order decline and was calculated from the reported group median viral densities at each measured timepoint in the first week following enrolment. If median viral loads were not reported or could not be derived, then the mean rate of viral clearance was calculated instead, using reported mean viral loads at each timepoint, or study-adjusted mean change in viral load from baseline (least-squares mean change). An effective antiviral should accelerate viral clearance in comparison with the contemporaneous untreated control group. The virological treatment effect is the viral clearance rate ratio (VCRR) between the no-treatment and active-treatment arms. The more effective the antiviral, the higher is the VCRR. Based on our own observations from over 800 patients with acute COVID-19 in whom detailed viral clearance estimates have been made,^18–20^ the variation in this ratio (per study) depending on whether means, study-adjusted means or medians were used was assumed to be small in comparison with the variation resulting from between-study sampling heterogeneity.

### Statistical methods

#### Primary analysis: evaluating the association between acceleration of SARS-CoV-2 clearance and the risk of hospitalization or death in randomized trials

A mixed-effects meta-regression model was used to quantify the association between the VCRR and the log relative risk of hospitalization or death in early symptomatic COVID-19.^7,21^ First, we summarized each randomized comparison in each study (intervention versus untreated control) by the relative risk (95% CI) for hospitalization or death. Comparisons in which a risk ratio could not be calculated (i.e. no events in one study arm) were not included in this analysis (see Table S5). Second, we summarized each randomized comparison in each study (intervention versus untreated control) by the median (or mean) VCRR. For RCTs with >1 experimental arm and one control group, the relative risk of hospitalization and/or death, and VCRRs were calculated for each experimental arm versus the control group. The log relative risk of hospitalization or death was the dependent (outcome) variable and VCRR was the fixed-effect independent variable. Each study was weighted by the inverse of the sampling variance of the hospitalization or death risk ratio plus the estimate of between-study heterogeneity (τ2). Between-study heterogeneity was estimated using the maximum-likelihood variance estimator. The linear association between the VCRR and log relative risk of hospitalization or death was quantified by the coefficient that determined the slope between these two variables. The overall variation in the risk ratio for hospitalization or death explained by the VCRR (i.e. the amount of between-study heterogeneity accounted for by the virological endpoint) was quantified by the regression coefficient of determination (R^2^). The higher the R^2^, the greater the proportion of the clinical treatment effect that is captured by the surrogate.

The following study subgroups were pre-specified: (i) RCTs including predominantly unvaccinated populations, i.e. <50% vaccinated; and (ii) RCTs including participants infected with predominantly pre-Delta SARS-CoV-2 variants of concern (VOCs) or completed enrolment before emergence of the Delta VOC.

The meta-regression analyses were performed in R using the metafor package.^22^

Risk of bias was assessed for both clinical and virological endpoints. The clinical endpoints were assessed using the Jadad scale,^23^ with a score out of five assessing the domains of randomization, blinding and description of withdrawals and dropouts. A score of ≥3 indicated a study was of high quality.

The viral load data quality was assessed with a maximum score of three; one point each was awarded for each of the following: (i) availability of viral density values at 3 or more timepoints between Days 0 and 8; (ii) reporting of viral densities as log_10_ copies/mL, or provision of Ct values with conversion formula to log_10_ copies/mL; and (iii) viral density data provided in a table or free text, i.e. not derived from a graph.

A score of two or higher was considered good-quality viral load data.

#### Secondary analyses

In a pre-specified secondary analysis, we compared the VCRRs between therapies that showed clinical efficacy in early symptomatic COVID-19 (including nMabs at the licensed doses and routes of administration that received emergency-use authorizations) and therapies not showing significant clinical efficacy in RCTs (Table 1).

**Table 1.** Pre-specified efficacy categories of therapies evaluated in trials of early symptomatic COVID-19

Clinically efficacious	Not clinically efficacious^a^	Uncertain clinical efficacy^b^
Remdesivir^5^ Nirmatrelvir/ritonavir^3^ Molnupiravir 800mg^2^ Ensitrelvir 125mg^24^ Ensitrelvir 250mg^24^ Sotrovimab^25^ Casirivimab/imdevimab 1200 mg and 2400mg^4^ IM Tixagevimab/cilgavimab^26^ Bamlanivimab 700mg^27^ Bamlanivimab/etesevimab 2800/2800 mg^28^ and 700/1400mg^13^ Bebtelovimab 175mg^29^ Regdanvimab 40 mg/kg^30^ Amubarvimab/romlusevimab^31^ Pegylated interferon lambda^32^ Convalescent plasma^33^	Hydroxychloroquine^34^ Ivermectin^35^ Favipiravir^36^ Lopinavir/ritonavir^37^ Nitazoxanide^38^ Fluvoxamine^39^	Tenofovir disoproxil fumarate/emtricitabine^40^ Artesunate/amodiaquine^41^ Pyronaridine/artesunate^41^ Sofosbuvir/daclatasivir^42^ Metformin^43^

^a^Includes combination therapies of ineffective drugs: favipiravir/nitazoxanide, hydroxychloroquine/azithromycin, favipiravir plus lopinavir/ritonavir.

^b^Insufficient or conflicting evidence.

In addition, we included three interventions where efficacy was suggested by meta-analyses and/or real-world effectiveness studies: the nMabs bebtelovimab and bamlanivimab 700 mg, which both received emergency-use authorizations and were effective in observational studies;^27,29^ and convalescent plasma. In the two convalescent plasma trials with viral load sampling included in this analysis, there was no significant reduction in hospitalization/death rates but an individual patient data (IPD) meta-analysis of five convalescent plasma trials did suggest moderate benefit.^33^

The following therapies were deemed to not have significant clinical efficacy based on evidence from RCTs, published systematic reviews and meta-analyses: hydroxychloroquine,^34^ ivermectin,^35^ favipiravir,^36^ lopinavir/ritonavir,^37^ nitazoxanide^38^ and fluvoxamine.^39^ Therapies were included in the ‘uncertain clinical efficacy’ category, where evidence of clinical efficacy in early symptomatic COVID-19 was sparse, based on small trials underpowered to detect clinical treatment effects, or evidence was conflicting (metformin^43^).

## Results

### Search results

**Figure 1.**
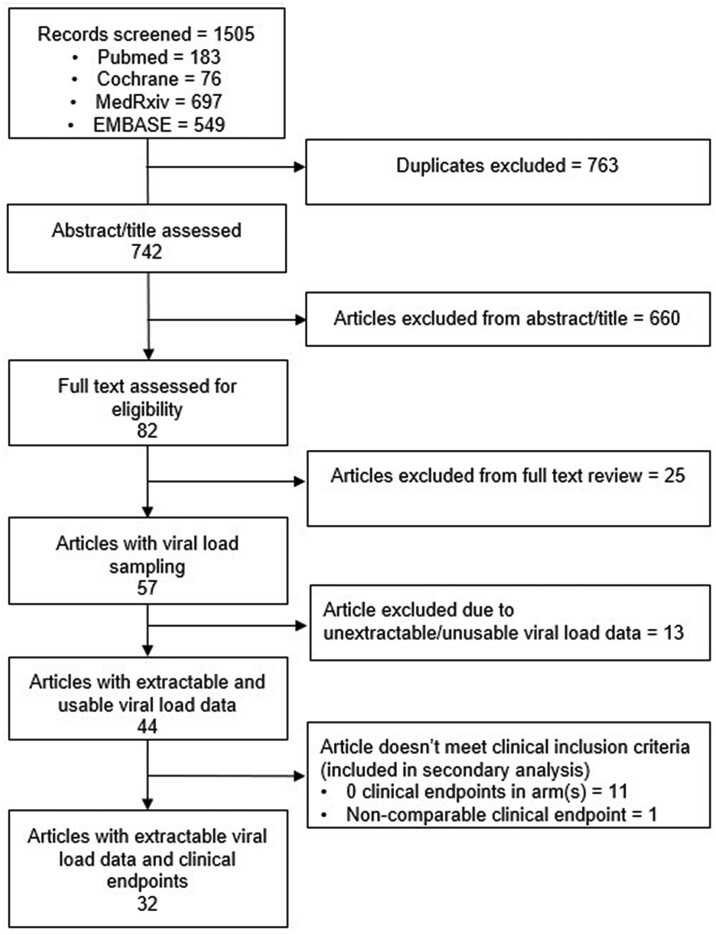
Consort diagram of search strategy and included trials.

The search strategy identified 57 studies (Figure 1) that met the eligibility criteria. Of these, 44 (77%) RCTs were eligible for analysis, i.e. with viral load data that could be used to derive a VCRR (Table S1). The 44 RCTs enrolled 52 384 participants and included 9 RCTs of small-molecule antivirals, 3 RCTs of pegylated interferon lambda, 15 RCTs of nMabs, 2 RCTs of convalescent plasma and 16 RCTs of repurposed therapies. One RCT included both remdesivir and casirivimab/imdevimab.^18^ Of these, 32 RCTs (73%; 49 313 participants) recorded at least one clinical endpoint (hospitalization and/or death) in each arm and were included in the meta-regression analysis. The 12 RCTs excluded from the meta-regression [excluding 3071 (5.9%) participants) had similar distributions of VCRRs (median 1.18, IQR 0.97–1.31, range 0.69–1.72), compared with the 32 included RCTs (median 1.18, IQR 1.07–1.34, range 0.69–1.79). All 44 RCTs were included in the secondary analysis.

Details of the excluded studies are provided in Supplementary methods 4(a and b).

### Baseline characteristics of included studies

Between March 2020 and October 2022, the 44 RCTs enrolled 52 384 participants; the median (IQR) number of participants enrolled per study was 300 (149–807). During this period there were successive waves of new SARS-CoV-2 variants and there was increasing vaccine coverage and acquired immunity so the trials differed with regard to infecting SARS-CoV-2 variants, the proportions of participants with risk factors for disease progression, patient previous exposures and vaccination status (Table 2) and thus progression to severe illness.

**Table 2.** Characteristics of analysed trials

Trial characteristic/baseline characteristics		Number of studies (*n*/*N*) or median value
Trial intervention	Small-molecule antivirals	9/44
	Pegylated interferon lambda	3/44
	Convalescent plasma	2/44
	Repurposed therapies	16/44
	Monoclonal antibodies	15^a^/44
Number of arms in each study	2	29/44
	3	4/44
	4	8/44
	5	2/44
	6	1/44
Randomization ratio	1:1 (50%)	36/68 arms
	1:0.818–1:1.22 (45%–55%)	60/68 arms
Number of participants enrolled per study	All 44 studies	300 (IQR 149–807, range 56–25 054)
	Meta-regression analysis (32 studies all patients)	519 (IQR 223–1138, range 60–25 054)
Median of the average age (years)		43 (IQR 36–48, range 27–60)
Median of the average symptom duration prior to enrolment (days)		4 (IQR 3–4.9, range 0–6)
Risk of progression to severe disease (% with risk factors based on US CDC/WHO criteria)	>90	14/44
	50–80	8/44
	<50	22/44
Infecting SARS-CoV-2 variant(s)	Pre-Delta	29/44
	Delta and/or Omicron	15/44
	Omicron only	3/44
Vaccination status (% receiving at least one dose)	≥50	9/44
	<50^b^	35/44
Serological status (% seropositive at baseline across 20 studies reporting these data)	≥50	16/20
	<50	4/20
	% Seropositive pre-Delta versus Delta/Omicron	13 (IQR 9.3–26.3) versus 51.2 (IQR 19.8–84)
Baseline pharyngeal viral swab eluate density, copies/mL (median of mean or median)	Intervention arms	1 000 000 copies/mL (IQR 112 202–3 630 781, range 4266–134 896 288)
	Control arms	851 138 copies/mL (IQR 97 724–4 265 795, range 1000–95 499 259)
Clinical endpoints (across 32 studies with ≥1 clinical endpoint in each arm included in the meta-regression analysis)	Reported all-cause hospitalization or death	24/32
	Reported COVID-19-related hospitalization or death	6/32
	Reported COVID-19-related ED visits or hospitalizations^c^	1/32
	Serious adverse events extracted as proxy for all-cause hospitalization or death	1/32
Sampling technique	Nasopharyngeal	26/44
	Oropharyngeal	7/44
	Nasal	9/44
	Saliva	2/44
RT–PCR assay LLOQ across 15 studies reporting this information (log_10_ copies/mL)	Range of LLOQ	2.3–3
	Range of values imputed for viral loads below LLOQ	1.7–3.27
Post-baseline viral load sampling (latest timepoint within the first 8 days post-randomization)	Day 7/8	33/44
	Day 5/6	10/44
	Day 4	1/44
Viral density data used for rate of clearance estimation	Median viral load data	20/44
	Mean viral load data	17/44
	Least-squares mean change from baseline	7/44

ED, emergency department; LLOQ, lower limit of quantification.

^a^One study included both remdesivir and casirivimab/imdevimab.^18^

^b^In 10 studies where baseline vaccination status was not reported, the patients were assumed to be predominantly unvaccinated based on the time when the study was conducted.

^c^Since 12 out of the 15 total ED visits/hospitalizations in the BLAZE-1 study were hospitalizations, this composite endpoint was considered largely comparable to the endpoints in other studies.

The following are included in the Supplementary data: eligible studies and their baseline characteristics (Tables S1 and S3); ineligible studies and their baseline characteristics (Tables S2 and S4); and clinical (Table S5) and virological outcomes (Table S6) for each eligible study.

The majority of ineligible studies were conducted in the pre-Delta variant period in unvaccinated participants, and the average age and symptom duration prior to enrolment were similar.

In each trial the intervention or interventions were compared with standard of care, which meant symptomatic treatment for an acute febrile illness. In two trials, therapies with potential COVID-19 antiviral efficacy, other than the experimental therapy, were used in control participants.^44,45^ In 10 of the 44 RCTs, at least a proportion of the trial was open label, and the remainder were placebo-controlled and blinded.

### Risk of bias

All studies scored 3 or higher in the Jadad scale for clinical endpoint bias, when assessed. The quality of the viral load data was also good, with scores of ≥2 in 36 of the 44 RCTs.

### Risk of hospitalization or death in control groups

Amongst the 25 462 patients enrolled across all control groups there were 794 (3.1%) hospitalizations or deaths (composite endpoint: median 5 per study, IQR 1–24, range 0–98). Of these 794 endpoints, 71 (9%) were deaths. The REGEN Phase 2 trial was excluded from this total as it combined all COVID-19-related medically attended visits into its clinical endpoint (including telemedicine visits, urgent care, in-person physician visits, ED visits and hospitalization).^4^ Hospitalization or death was higher amongst trials (*n* = 35) that enrolled predominantly unvaccinated patients than in trials enrolling predominantly vaccinated patients (*n* = 9): median 5.7% (IQR 2.9%–7.5%, range 0%–11.2%) versus 1.5% (IQR 1.0%–2.5%, range 0%–4.4%). Hospitalization or death was higher in trials (*n* = 29) conducted before the Delta VOC wave (median 5.7%, IQR 3.3%–7.1%) than in later trials (median 1.6%, IQR 0.8%–4.3%; *n* = 15).

A summary of hospitalizations and deaths stratified by risk (risk of progression to severe disease by original WHO/CDC criteria) and vaccination status is shown in Table 3.

**Table 3.** Proportion of hospitalizations/deaths and deaths across control groups stratified by risk and vaccination status

	Unvaccinated, hospitalized/died *n*/*N* (%)	Died *n*/*N* (%)	Vaccinated, hospitalized/died *n*/*N* (%)	Died *n*/*N* (%)
Predominantly low risk	55/1534 (3.6)	1/1534 (0.07)	9/626 (1.4)	0/626 (0.0)
Predominantly high risk	564/8401 (6.7)	61/8401 (0.7)	166/14 901 (1.1)	9/14 901 (0.06)

### Viral clearance rates in control groups

**Figure 2.**
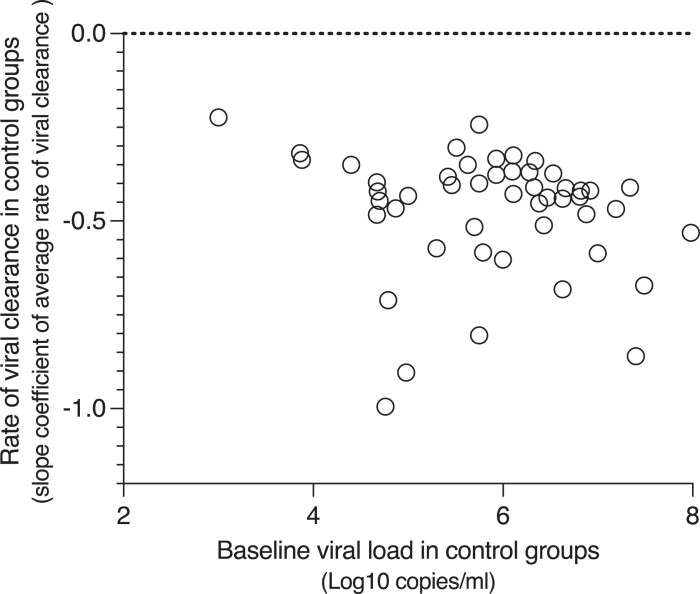
The relationship between the baseline mean/median viral densities and rates of viral clearance (log_10_ viral copies/mL/day) among the control (no treatment) groups.

The median viral clearance rate estimate across control (untreated) groups was −0.43 (IQR −0.52 to −0.37, range −1 to −0.22) log_10_ viral copies/mL/day. Viral clearance was faster in studies conducted during the later Delta/Omicron waves compared with earlier studies (median −0.53 versus −0.42; *P* = 0.015). There was no difference between the vaccinated and unvaccinated groups (median −0.45 versus −0.42; *P* = 0.2). There was a negative but non-significant correlation between baseline viral densities and the rates of viral clearance in the control groups (Spearman’s rank correlation coefficient = −0.25, 95% CI −0.51 to 0.04; *P* = 0.08, Figure 2).

### Association between rates of hospitalization and/or death and SARS-CoV-2 pharyngeal VCRRs

**Figure 3.**
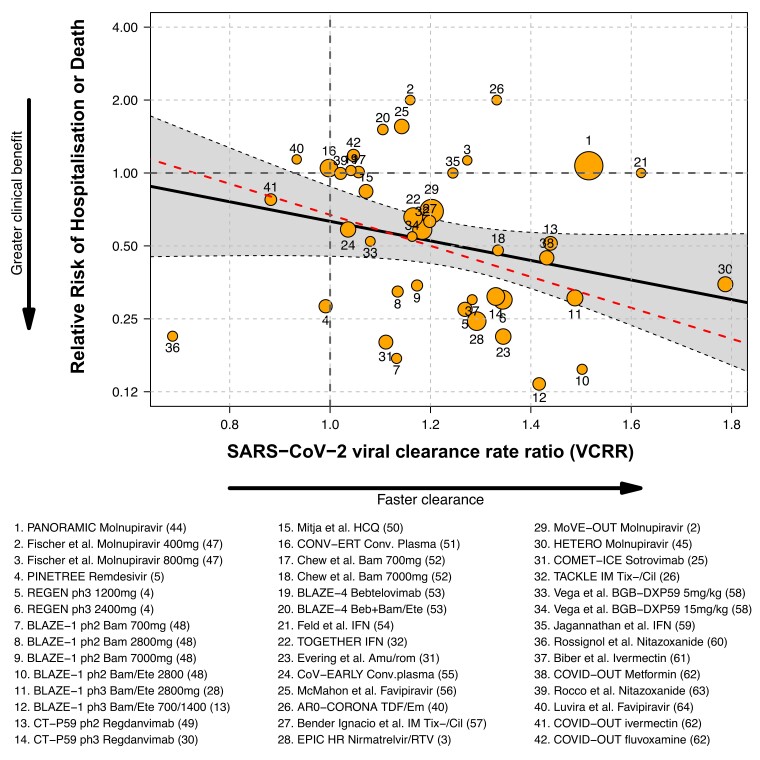
Meta-regression analysis of the relationship between SARS-CoV-2 rate of clearance relative to control (VCRR), and relative risk of hospitalization/death. Each study is labelled with a number (Table S7). The area of the circles is proportional to the inverse of the standard error of the clinical effect size. Solid black line: regression analysis including all 32 eligible studies.^2–5,13,25,26,28,30–32,40,44,45,47–64^ Red dashed line: exclusion of the large PANORAMIC study (label 1).^25^

Including all trials eligible for the meta-regression analysis, 9.7% (R^2^) of the variation in the risk ratios for severe outcomes was explained by variation in virological effects (VCRR), with a non-significant association between the clinical and virological treatment effects [slope = −0.92 (95% CI −1.99 to 0.13); *P* = 0.08]. However, this estimate was highly sensitive to the inclusion of a single trial. Influential case diagnostics (Cook’s distance^46^) identified the very large PANORAMIC trial,^44^ (Figure 3, label 1) as having the most influence on the overall estimated regression coefficient. PANORAMIC was a later study of molnupiravir in a highly vaccinated high-risk outpatient population in which a large acceleration in viral clearance was observed (VCRR: 1.52) in the small subgroup (*n* = 73; 0.3%) of participants recruited at the end of the study who agreed to conduct daily virology self-swabbing. There was no reduction in the risk of hospitalization or death in the PANORAMIC trial [relative risk 1.07 (95% CI 0.8–1.4); Table S7].^44^ Omitting this single outlier results in a marked increase in R^2^ to 50.4%, with a statistically significant association between VCRR and risk ratios for severe outcomes [slope −1.47 (95% CI −2.43 to −0.51); *P* = 0.003]; higher VCRRs were associated with lower risks of severe outcomes (Figure 3, represented by red dashed line).

We investigated whether vaccination status (<50% versus ≥50%) or variant status (pre-Delta versus Delta and/or Omicron) modified the association between the virological predictor and the clinical treatment effect, through interaction analysis. Neither of these additional covariates were identified as clear effect modifiers (Table S8).

A sensitivity analysis was performed in which those studies with no clinical endpoints in one arm were included in the meta-regression, assigning 0.5 for an event instead of 0. This generated similar results to those of the main analysis [R^2^ = 9.9%; meta-regression slope −1.0 (95% CI −1.98 to −0.03); *P* = 0.04].

### Comparing viral clearance rates between clinically efficacious and non-efficacious therapies

**Figure 4.**
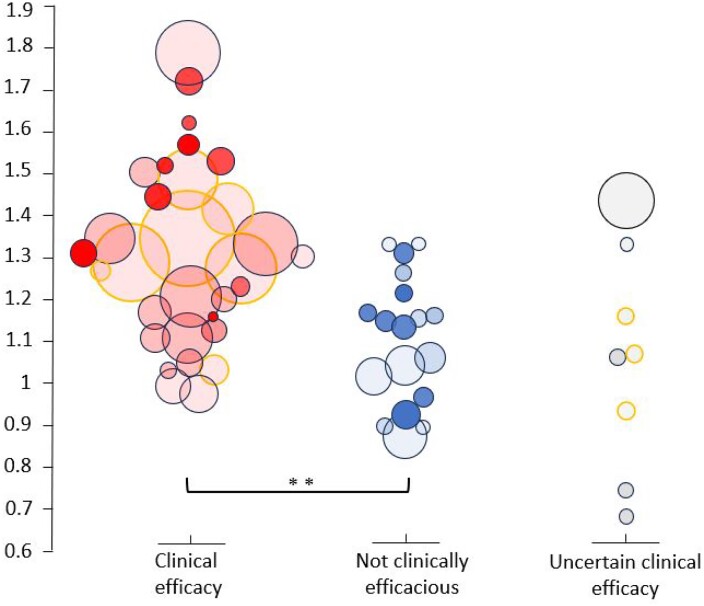
Comparison of rates of pharyngeal clearance of SARS-CoV-2 relative to control/placebo between therapies that have shown clinical efficacy, those that have not, and those where the effect is uncertain. Circle size is proportional to the square root of the total virological sample size at baseline. Intensity of shading is proportional to the frequency of viral load sampling. Studies for which viral clearance rates were calculated using the least-squares mean change from baseline (adjusted for baseline viral load and other covariates) are indicated by the orange outline.

The VCRR values were greater for therapies that have shown strong evidence of clinical efficacy in Phase 3 trials or meta-analyses, compared with therapies that have not (median VCRR 1.29 versus 1.14; *P* = 0.001) (Figure 4). The ‘not clinically efficacious’ group included a higher proportion of studies with highly vaccinated patients (≥50%) compared with the ‘clinical efficacy’ group (37% versus 19%), and a higher proportion of studies conducted in the Delta and/or Omicron period (47% versus 35%).

Amongst studies of clinically efficacious interventions, the VCRRs were similar between studies with predominantly vaccinated patients (≥50%) and studies with predominantly unvaccinated patients (median VCRR: 1.42 versus 1.27; *P* = 0.3). The VCRRs were also similar in studies conducted in the pre-Delta period compared with studies conducted in the Delta and/or Omicron period (median VCRR: 1.27 versus 1.31; *P* = 0.2).

## Discussion

COVID-19 continues to cause a significant burden of disease, particularly in vulnerable subgroups. New SARS-CoV-2 variants are constantly emerging. Effective medicines are still needed. Although most repurposed medicines were ineffective in the treatment of early COVID-19 infections, specific antiviral monoclonal antibodies and new small-molecule drugs have proved efficacious in large RCTs in preventing COVID-19 progression to hospitalization and death. But the severity of COVID-19 has declined substantially over the past 4 years. At the beginning of the pandemic, previously healthy individuals often developed severe respiratory compromise, whereas today severe infection and death is confined largely to those with underlying conditions, or the elderly and frail. In the majority of patients without multiple comorbidities (i.e. low-risk individuals) the clinical benefits of an effective antiviral are less. Whereas, earlier in the pandemic, RCTs that enrolled several hundreds of patients had sufficient statistical power to identify moderate but clinically important therapeutic benefits, this is no longer the case. Now trials that enrolled many thousands of patients would still lack sufficient power to identify moderate treatment effects because of the low risk of disease progression requiring hospitalization. But COVID-19 can still be a trigger for hospital admission in high-risk patients or a contributor to a terminal illness in the frail, debilitated or elderly. Amongst these vulnerable patients there are some in whom viral pathogenesis is the predominant problem, and who will benefit substantially from effective antiviral drugs, just as patients did earlier in the pandemic.

This meta-analysis shows that when omitting the recent large PANORAMIC trial,^44^ there is a clear relationship between clinical efficacy, measured by prevention of hospitalization and death, and rates of viral clearance (VCRRs) across all RCTs of antiviral therapies. This finding is supported by two previous meta-analyses.^6,7^ The PANORAMIC trial, which was conducted in a highly vaccinated, high-risk outpatient population is a clear outlier in this meta-analysis. PANORAMIC contributed over half the patients and 16.6% of the endpoints in the analysis, but measured viral reduction in <2%, and viral clearance rates in only 0.3% of the enrolled patients. Molnupiravir accelerated viral clearance substantially, as in more detailed evaluations,^19^ and was associated with significantly lower Day 5, but higher Day 14 viral densities. In the overall study (*n* = 26 411), molnupiravir treatment resulted in earlier sustained recovery, higher self-rated wellness, and reductions in times to sustained recovery, times to alleviation of all symptoms, times to sustained alleviation of all symptoms, and times to reduction of symptom severity. Molnupiravir was also associated with fewer moderate or severe symptoms at Days 7, 14 and 28, and less contact with GPs, but it did not reduce the low rates of hospitalization and/or death.^44^ In the combined analysis of all the other studies, acceleration in viral clearance accounted for approximately half of the variance in progression to severe disease. Interaction analysis (including the PANORAMIC trial) demonstrated that the association between the virological and clinical endpoints was not dependent on vaccination or variant status, i.e. a consistent association was seen in unvaccinated and vaccinated subgroups and in pre-Delta and Delta/Omicron subgroups. Our secondary analysis also indicates that the VCRRs were higher in trials of efficacious interventions compared with trials of non-efficacious interventions.

Characterizing pharyngeal viral clearance adequately requires frequent sampling as the measure is inherently very ‘noisy’. But most studies in this meta-analysis measured only two or three sequential samples so the estimates are inaccurate. Large intra-subject variance is not surprising considering that the quantitative PCR is performed on viral nucleic acid extracted from a viral transport medium in which a swab has been immersed. The process of swabbing varies considerably, the quantity of virus in the pharynx is heterogeneous, and the amount of material on the swab is also highly variable. Repeated nasopharyngeal sampling is tolerated poorly. Some patients present while virus densities in the pharynx are still rising, and others present after densities have begun to fall. A further issue is that the true viral elimination profile is biphasic (biexponential), and samples collected on Day 7 (a common sampling time) are often well after the end of the first phase of elimination.^20^ As a consequence, the ‘drug-sensitive’ first phase of elimination is systematically underestimated, and so the VCRR is reduced.^20^ A recent meta-analysis of trials in unvaccinated patients found that treatment-induced acceleration of viral clearance measured within 5 days, but not at 7 days, was associated with decreased odds of progression to hospitalization or death.^6^ Our own individual patient data analysis, which now includes over 1300 detailed individual series, shows that viral clearance rates have accelerated substantially over the past 2 years and, as a consequence, VCRRs estimated over 5 days have shown progressively greater discriminant value compared with 7 days.^20^

This systematic review has several important limitations. It is dominated by the recent (December 2021 to April 2022) large PANORAMIC study,^44^ which is a highly influential outlier in this assessment of the relationship between viral clearance and clinical benefit. For most studies, summary viral load data rather than individual patient data were used to derive viral clearance rates. Thus, it was not possible to derive an uncertainty measure for the virological treatment effects. The substantial inter- and intra-subject variances make viral clearance assessments based only on one or two post-treatment samples very inaccurate. This is likely to be a major contributor to the failure to establish a relationship between viral clearance and risk of hospitalization and death in some studies. It still remains unclear why there is so much inter-individual variability (i.e. differences in virus clearance rates between people). Immune status is likely to be an important determinant. In addition, the trials were very different. Although date and location (reflecting both prevalent viruses and immune status) are clearly very important, the study populations also varied in terms of risk factors, allowed duration of illness before enrolment, and criteria for hospitalization. There were substantial differences in sampling schedules, methods of statistical adjustment and reporting, and methods for dealing with negative post-treatment PCRs. There are also systematic errors in the method of viral clearance assessment described above (particularly affecting studies with only a Day 0 and Day 7 measurement, which systematically underestimates the rate of the initial phase of viral clearance). Finally, we could not obtain non-published data from all the relevant therapeutic studies so we cannot say if these would have affected our estimates.

Interventions that proved effective in RCTs were associated with accelerated viral clearance whereas those that did not show clinical benefit usually did not accelerate viral clearance. Although this systematic overview supports measurement of viral clearance as a surrogate for clinical efficacy, there remains uncertainty. This emphasizes the importance of standardizing the viral clearance assessment methodology so that different studies can be compared more effectively than in this, and earlier, retrospective studies.^6,7^ Provision of raw data for independent patient-data meta-analysis would be much better than use of individual study summary metrics. Recent studies suggest that daily sampling for 5 days would be adequate with currently prevalent SARS-CoV-2.^20^ Repeated oropharyngeal sampling is much better tolerated than repeated nasopharyngeal sampling, thereby allowing studies to take more samples, and it provides comparable results. Standardized methods of assessing viral clearance can be used to assess and compare antiviral drugs for COVID-19, and probably most other acute respiratory viral infections.^19^ We would also like it.

## Supplementary Material

dkae045_Supplementary_Data

## Supplementary data

Supplementary methods and Tables S1 to S8 are available as Supplementary data at *JAC* Online.
