# Supplementary material for: The relationship between viral clearance rates and disease progression in early symptomatic COVID-19: a systematic review and meta-regression analysis
Source: J Antimicrob Chemother. 2024 Feb 22;79(5):935–45. doi: 10.1093/jac/dkae045 (PMC11062948; doi:10.1093/jac/dkae045)
Supplement: dkae045_Supplementary_Data [file dkae045_supplementary_data.docx]

**Supplementary appendix**

1. **Search Strategy**

A literature search of PubMed, EMBASE, *MedRxiv*, and Cochrane Reviews for studies satisfying the inclusion/exclusion criteria was conducted. The search strategy was designed in collaboration with the Bodleian Health Care librarian at the University of Oxford. The following keywords were used in several combinations – coronavirus disease, severe acute respiratory syndrome coronavirus 2, coronavirus, 2019nCoV, SARS CoV-2*, severe acute respiratory syndrome, COVID, mild, moderate, outpatient, randomi*ed, paxlovid, nirmatrelvir, molnupiravir, remdesivir, ensitrelvir, S-217622, ivermectin, lopinavir, hydroxychloroquine, chloroquine, fluvoxamine, fluoxetine, favipiravir, interferon-lambda, peginterferon lambda, nitazoxanide, monoclonal antibod*, sotrovimab, REGN-COV, REGEN-COV, imdevimab, casirivimab, evusheld, tixagevimab, cilgavimab, bamlanivimab, etesevimab, bebtelovimab, regdanvimab, romlusevimab. The final search was run on 18^th^ July 2023.

After removing duplicates, two reviewers independently identified papers for full text screening (S.S, S.B), with any discrepancies resolved by a third reviewer (W.S).

1. **Data extraction**

*Clinical and demographic details*

A standardised form was used to extract data from the included studies. Data extraction was performed independently by dual entry (S.S, S.B). Discrepancies were resolved through discussion, with review by a third investigator if necessary. The following parameters were extracted from included studies: trial design (open label or blind, single or multicentre), entry criteria, dates of start and end of recruitment, geographical region(s), baseline clinical and demographic characteristics of total population and by study arm (unselected or patients with a high-risk of disease progression, vaccination status, age, duration of symptoms at randomisation, infecting variants, serology status, baseline pharyngeal viral density), definition of primary endpoint, viral sampling method and lower limits of quantification/lower limit of detection of qPCR assay, if reported. Clinical outcomes extracted were the number of participants with all-cause (or COVID-19 related if all-cause not reported/could not be obtained from authors) hospitalisation or all-cause death in each study arm.

1. **Viral loads**

Serial median pharyngeal swab eluate viral densities (Ct or Log10 RNA copies/ml) were extracted for the intervention and control arms. Densities were either extracted as numerical values from tables, or from viral density (or mean change in viral density) vs. time graphs using Web Plot Digitizer.^1^ If group median viral density values were not reported, we contacted the study authors to request this information. For studies where the authors did not reply after several attempts, we extracted reported mean viral loads. Where individual patient data were provided by authors, participants with undetectable viral loads at all timepoints (i.e., who were not infected), were excluded before calculating median viral loads. Clinical and viral load data from the overall population were extracted where the analysis of a subgroup was reported as the primary clinical and/or virologic outcome. If viral loads were reported as Ct values without a Log10 RNA copies/ml conversion formula, Ct values were converted to the Log10 copies/ml scale using the formula 45-Ct/Log_2_10 (the conversion formula used in the BLAZE-1 study).^2^

1. **Excluded studies**
2. *Exclusion from all analyses (supplementary Table 2)*

Thirteen of 57 eligible studies (23%) did not have extractable viral load data and their data were not provided by authors despite e-mail requests, and so these studies could not be included in the analysis. The baseline characteristics of these studies were largely comparable with those included (table S4), but reported less vaccination, seropositivity and baseline viral load data. One of the thirteen studies excluded (Omrani *et al*)^3^ reported median Ct values at baseline and change in Ct at day 6, but the change in Ct was calculated only for participants not achieving virologic cure by day 6 i.e., it did not evaluate those patients clearing virus earlier. As median day 6 Ct values were not provided by the authors, this study was excluded.

1. *Exclusion from the meta-regression*

11 out of 44 RCTs were excluded from the meta-regression analysis because there were no clinical endpoints in at least one treatment arm. The RCT by Weinreich et al (REGEN phase 2)^4^ was also excluded as they combined all COVID-19 related medically attended visits (including telemedicine visits, urgent care, in-person physician visit, ED visit, hospitalisation) into one composite endpoint and did not report hospitalisations separately. This combined end-point was not considered comparable to hospitalisation or death endpoints. Thus 12 of 44 RCTs in total were excluded from the meta-regression.

**Table S1: Eligible studies included in review**

| **Author** | **Study** | **Year** | **Design** | **Therapy** | **Class** | **Primary endpoint(s)** |
| --- | --- | --- | --- | --- | --- | --- |
| Gottlieb^5^ | PINETREE | 2021 | Phase 3 double-blind | Remdesivir | Antiviral | COVID-19–related hospitalisation/all-cause death by day 28 |
| Weinreich^4^ | REGEN | 2021 | Phase 2 double-blind | Casiri-/imdevimab 2400mg  Casiri-/imdevimab 8000mg | mAb | Change in viral load from baseline to day 7 |
| Weinreich^6^ | REGEN1200+2400 | 2021 | Phase 3 double-blind | Casiri-/imdevimab 1200mg  Casiri-/imdevimab 2400mg | mAb | COVID-19–related hospitalisation/all-cause death by day 28 |
| Parienti^7^ | AR0-CORONA | 2021 | Phase 2 open-label | Tenofovir disoproxil fumarate/emtricitabine | Repurposed | Change in viral load from baseline to day 4 |
| Feld^8^ | ILIAD | 2021 | Phase 2 double-blind | Interferon lambda-1a | Type III Interferon | Proportion with negative RT-PCR on day 7 |
| Montgomery^9^ | TACKLE | 2022 | Phase 3 double-blind | Tixa-/cilgavimab IM 600mg | mAb | Severe COVID-19 (pneumonia or hypoxemia plus WHO Clinical Progression Scale score of 5 or more)/all-cause death by day 29 |
| Reis^10^ | TOGETHER | 2023 | Phase 3 double-blind | Interferon lambda-1a | Type III Interferon | COVID-19–related ER visit >6 hours/hospitalisation by day 28 |
| Dougan^11^ | BLAZE-4 | 2022 | Phase 2 double-blind | Bebtelovimab  Beb-/bam-/etesevimab | mAb | Persistently high viral load at day 7 |
| Chew^12^ | ACTIV-2 | 2022 | Phase 2 double-blind | Bamlanivimab 700mg  Bamlanivimab 7000mg | mAb | Proportion with negative RT-PCR at days 3/7/14/21/28 |
| Alemany^13^ | CONV-ERT | 2022 | Phase 2 double-blind | Methylene-blue treated convalescent plasma | Conv.Plasma | All-cause hospitalisation by day 28  Change in viral load from baseline to day 7 |
| Gharbharan^14^ | CoV-Early | 2023 | Phase 3 double-blind | Convalescent plasma | Conv.Plasma | Clinical progression (measured by 5-point ordinal scale) by day 28 |
| Evering^15^ | ACTIV-2 | 2023 | Phase 3 double-blind | Amubarvimab/romlusevimab | mAb | All-cause hospitalisation/death by day 28  Grade 3 or higher treatment-emergent adverse effects by day 28 |
| Bender Ignacio^16^ | ACTIV-2 | 2023 | Phase 2 double-blind | Tixa/cilgavimab IM Tixa/cilgavimab IV | mAb | Proportion with negative RT-PCR at days 3/7/14  Time to symptom improvement by day 28  Grade 3 or higher treatment-emergent adverse effects by day 28 |
| Bernal^17^ | MoVE-OUT | 2022 | Phase 3 double-blind | Molnupiravir 800mg | Antiviral | All-cause hospitalisation/death by day 29 |
| Butler^18^ | PANORAMIC | 2023 | Phase 3 open-label | Molnupiravir 800mg | Antiviral | All-cause hospitalisation/death by day 28 |
| Gottlieb^19^ | BLAZE-1 | 2022 | Phase 2 double-blind | Bamlanivimab 700mg  Bamlanivimab 7000mg  Bamlanivimab 2800mg  Bamlanivimab + etesevimab 2800/2800mg | mAb | Change in viral load from baseline to day 11 |
| Gupta^20^ | COMET-ICE | 2022 | Phase 3 double-blind | Sotrovimab | mAb | All-cause hospitalisation/death by day 29 |
| Biber^21^ |  | 2022 | Double-blind | Ivermectin | Antiviral | Proportion with negative RT-PCR on day 6 |
| Sinha^22^ | HETERO | 2022 | Phase 3 open-label | Molnupiravir 800mg | Antiviral | All-cause hospitalisation/death by day 14 |
| Mitja^23^ | BCN PEP-CoV-2 | 2021 | Phase 3 open-label | Hydroxychloroquine | Antiviral | Change in viral load from baseline to days 3 and 7 |
| Jagannathan^24^ | COVID-Lambda | 2021 | Phase 2 single-blind | Interferon lambda-1a | Type III Interferon | Time to viral clearance (first of two consecutive negative RT-PCRs) |
| Kim^25^ | CT-P59 | 2022 | Phase 3 double-blind | Regdanvimab 40mg/kg | mAb | COVID-19 disease progression (hospitalization/oxygen therapy/mortality) by day 28 among “high-risk” patients |
| McMahon^26^ |  | 2022 | Phase 2 double-blind | Favipiravir | Antiviral | Time to viral clearance (first of two consecutive negative RT-PCRs) |
| Hammond^27^ | EPIC-HR | 2022 | Phase 2/3 double blind | Nirmatrelvir | Antiviral | COVID-19–related hospitalisation/death at day 28 in patients with ≤3 days symptom duration |
| Fischer^28^ |  | 2022 | Phase 2 double-blind | Molnupiravir 200mg  Molnupiravir 400mg  Molnupiravir 800mg | Antiviral | Time to viral clearance (first of two consecutive negative RT-PCRs) |
| Dougan^29^ | BLAZE-1 | 2021 | Phase 2/3 double blind | Bam-/etesevimab 2800/2800 | mAb | COVID-19 related hospitalisation/all-cause death by day 29 |
| Dougan^2^ | BLAZE-1 | 2021 | Phase 2/3 double blind | Bam-/etesevimab 700/1400 | mAb | COVID-19 related hospitalisation/any cause death by day 29 |
| Streinu-Cercel^30^ | CT-P59 | 2021 | Phase 2 double-blind | Regdanvimab 40mg/kg Regdanvimab 80mg/kg | mAb | Time to viral clearance (first of two consecutive negative RT-PCRs)  Time to clinical recovery (all symptoms scored as “absent” or “mild” for ≥24h) by day 14 |
| Vega^31^ |  | 2023 | Phase 2 double-blind | BGB-DXP593 5mg/kg  BGB-DXP593 15mg/kg  BGB-DXP593 30mg/kg | mAb | Change in viral load from baseline to day 8 |
| Rossignol^32^ |  | 2022 | Double-blind | Nitazoxanide | Repurposed | Time to sustained clinical recovery (measure of within-participant symptom improvement) within 28 days |
| Bramante^33^ | COVID-OUT | 2023 | Phase 3 quadruple blind | Metformin  Ivermectin  Fluvoxamine | Repurposed | Severe COVID-19 (hypoxaemia/ED visit/ hospitalisation/death) by day 14 |
| Rocco^34^ | SARITA-2 | 2022 | Double-blind | Nitazoxanide | Repurposed | Complete resolution of symptoms (dry cough, fever and/or fatigue) by day 14 |
| Luvira^35^ | PLATCOV | 2023 | Phase 2 open-label | Favipiravir | Repurposed | Rate of viral clearance |
| Mukae^36^ |  | 2023 | Phase 2b double-blind | Ensitrelvir 125mg  Ensitrelvir 250mg | Antiviral | Change in viral load from baseline to day 4  Change from baseline in COVID-19 symptoms |
| Khoo^37^ |  | 2022 | Phase 2 double-blind | Molnupiravir 800mg | Antiviral | Time to viral clearance (first of two consecutive negative RT-PCRs) |
| Holubar^38^ |  | 2022 | Phase 2 double-blind | Favipiravir | Repurposed | Time to viral clearance (first of two consecutive negative RT-PCRs) |
| Lowe^39^ | FLARE | 2022 | Phase 2, 2x2 factorial, double-blind | Favipiravir,  Lopinavir-ritonavir,  Favipiravir plus lopinavir-ritonavir | Repurposed  Repurposed  Repurposed | Change in viral load from baseline to day 5 |
| Sirijatuphat^40^ |  | 2022 | Open-label | Favipiravir | Repurposed | Time to clinical improvement (reduction in NEWS score from baseline or a score of ≤1) within 28 days |
| Rodrigues^41^ |  | 2021 | Double-blind | Hydroxychloroquine + azithromycin | Repurposed | Time to viral clearance (first of two consecutive negative RT-PCRs) |
| Buonfrate^42^ | COVER | 2022 | Phase 2 double-blind | Ivermectin 1200 μg/kg  Ivermectin 600 μg/kg | Repurposed | Incidence of serious adverse drug reactions  Change in viral load from baseline to day 7 |
| De la Rocha^43^ |  | 2022 | Double-blind | Ivermectin 12mg | Repurposed | Progression to severe COVID-19 (hypoxaemia, RR>30, lung infiltrates >50%) by day 14 |
| Chandiwana^44^ |  | 2022 | Phase 2 double-blind | Artesunate-amodiaquine  Pyronaridine-artesunate  Favipiravir plus nitazoxanide  Sofosbuvir-daclatasvir | Repurposed  Repurposed  Repurposed  Repurposed | Proportion with negative RT-PCR on day 7 |
| Schilling^45^ | PLATCOV | 2023 | Phase 2 open-label | Ivermectin 600 µg/kg | Repurposed | Rate of viral clearance |
| Jittmala^46^ | PLATCOV | 2022 | Phase 2 open-label | Remdesivir  Casiri-/imdevimab 1200mg | Repurposed  mAB | Rate of viral clearance |

**Table S2: Non-eligible studies**

| **Author** | **Study** | **Year** | **Design** | **Therapy** | **Class** | **Primary endpoint** |
| --- | --- | --- | --- | --- | --- | --- |
| Tippabhotla^47^ |  | 2022 | Phase 3 open-label | Molnupiravir 800mg | Antiviral | All-cause hospitalisation by day 14 |
| Bosaeed^48^ |  | 2022 | Double-blind | Favipiravir | Repurposed | Time to viral clearance (first of two consecutive negative RT  PCRs) |
| Golan^49^ | PRESECO | 2022 | Double-blind | Favipiravir | Repurposed | Time to sustained clinical recovery |
| Reis^50^ | TOGETHER | 2021 | Platform trial, double  blind where feasible | Hydroxychloroquine  Lopinavir-ritonavir | Repurposed | COVID-associated hospitalisation and death by day-90 |
| Reis^51^ | TOGETHER | 2022 | Platform trial, double-blind | Fluvoxamine | Repurposed | COVID-related ER visit >6hr or hospitalization by day 28 |
| Reis^52^ | TOGETHER | 2022 | Platform trial, double blind | Ivermectin | Repurposed | COVID-related ER visit >6hr or hospitalization by day 28 |
| Reis^53^ | TOGETHER | 2022 | Platform trial, double blind | Metformin | Repurposed | COVID-related ER visit >6hr or hospitalization by day 28 |
| Reis^54^ | TOGETHER | 2023 | Platform trial, double blind | Fluvoxamine and inhaled budesonide | Repurposed | COVID-related ER visit >6hr or hospitalization by day 28 |
| Johnston^55^ | COVID-19 Early | 2021 | Double-blind | Hydroxychloroquine  Hydroxychloroquine+  azithromycin | Repurposed | Progression to lower respiratory tract infection by day 14  COVID-19 related hospitalization or death by day 28  Time to viral clearance (first of two consecutive negative RT- PCRs) |
| Spivak^56^ |  | 2023 | Phase 2 double-blind | Hydroxychloroquine | Repurposed | Time to viral clearance (first of two consecutive negative RT PCRs) |
| Vallejos^57^ | IVERCOR-COVID | 2021 | Double-blind | Ivermectin | Repurposed | All-cause hospitalization within 44 days |
| Rezai^58^ |  | 2022 | Double-blind | Ivermectin | Repurposed | Time to resolution of symptoms |
| Omrani^3^ | Q-PROTECT | 2020 | Double-blind | Hydroxychloroquine  Hydroxychloroquine+  azithromycin | Repurposed | Proportion with negative RT-PCR on day 6 |

**Table S3: Baseline characteristics of eligible studies**

| **Author** | **Geography** | **Enrolment period** | **Infecting variants*** | **Average age (years)** | **Risk status** | **Average symptom duration (days)** | **% Vaccinated** | **% Sero-positive** | **Baseline VL drug (Log10 copes/ml)** | **Baseline VL control (Log10 copies/ml)** |
| --- | --- | --- | --- | --- | --- | --- | --- | --- | --- | --- |
| Chew  Bam 700mg^12^ | Worldwide | Aug-Nov2020 | Pre-delta | 47 | Unselected -51.5% high-risk | 6 | 0 | Not reported | 5.5 | 5.7 |
| Chew  Bam 7000mg^12^ | Worldwide | Aug-Nov2020 | Pre-delta | 45 | Unselected- 41.5% high-risk | 6 | 0 | Not reported | 5.2 | 5 |
| Alemany^13^ | Spain | Nov 2020 - July 2021 | Pre-delta (<5% delta) | 56 | Unselected – ~74% high-risk | 4.4 | 0 | Not reported | 7.21^∞^ | 7.19^∞^ |
| Gharbaran^14^ | The Netherlands | Nov 2020-July 2021 | Pre-delta (alpha and D614G dominant) | 60 | High-risk enrolled | 5 | 2.9 | 7.9 | 7.27^ | 7^ |
| Evering ^15^ | Worldwide | Jan-July 2021 | Pre-delta (77.8% pre-delta, 22.2% delta) | 49 | High-risk enrolled | 6 | 8.2 | 29 | 3.63 | 3.88 |
| Bender Ignacio^16^  IM Tixa-/Cil | US | Feb-May 2021 | Pre-delta (0.5% delta) | 39 | Unselected- 29.6% high risk | 6 | 6 | Not reported | 5.43 | 4.67 |
| Bender Ignacio^16^  IV Tixa-/Cil | US | Feb-May 2021 | Pre-delta (0.5% delta) | 44 | High-risk enrolled | 6 | 0.9 | Not reported | 3.91 | 3 |
| Bernal^17^ | Worldwide | May-Oct 2021 | Majority delta (58.1%) | 43 | High-risk enrolled | 3 | 0 | 19.8 | 6.81 | 6.81 |
| Butler  PANORAMIC^18^ | UK | Dec 21-April 22 | Majority omicron (99%) | 57 | High-risk enrolled | 2 | 99 | Not reported | 7.27 | 7.34 |
| Gottlieb  PINETREE^5^ | Worldwide | Sep 20 -April 21 | Pre-delta | 50 | High-risk enrolled | 5 | 0 | Not reported | 6.3 | 6.28 |
| Gupta^20^ | Worldwide | Aug 20-March 21 | Pre-delta | 53 | High-risk enrolled | 3 | Not reported^#^ | Not reported | 6.61 | 6.82 |
| Biber^21^ | Israel | May 20- Jan 21 | Pre-delta | 36 | Unselected – 13.7% high risk | 4 | Not  reported^#^ | Not reported | 6.32^ | 6.92^ |
| Gottlieb  BLAZE-1^19^ | US | June -Oct 20 | Pre-delta | 44 | Unselected- ~67% high risk | 4 | 0 | Not reported | Bam 700  6.38  Bam 2800  6.17  Bam 7000  6.5  B/E 2800  6.71 | 6.38 |
| Sinha^22^ | India | May-Aug 2021 | Delta period | 35 | Unselected | 3 | Not  reported | Not reported | 5.75 | 5.75 |
| Mitja^23^ | Spain | March-May 20 | Pre-delta | 41.5 | Unselected- ~53% high-risk | 3 | 0 | Not reported | 8.13 | 7.98 |
| Jagannathan^24^ | US | April-July 20 | Pre-delta | 36 | Unselected- median 3 risk factors | 5 | 0 | 40.8 | 3.92^∞^ | 3.86^∞^ |
| McMahon^26^ | Australia | July 2020-Sep 2021 | Highest enrolment Wuhan+delta | 36 | Unselected | Not  reported | Not  reported | Not reported | 6.11^∞^ | 5.75^∞^ |
| Kim^25^ | Worldwide | Jan 21- Apr 21 | Pre-delta (majority alpha) | 48 | Unselected –  64% high-risk | 4 | 0 | 11.3 | 6.07 | 6.1 |
| Feld^8^ | Canada | May-Sep 20 | Pre-delta | 44 | Unselected - ~15% high-risk | 4.5 | 0 | 10.5 | 6.16 | 4.87 |
| Reis  TOGETHER – Interferon^10^ | Brazil,  Canada | June 21 -Feb 22 | Majority delta (45%)+ omicron(41% | 43 | High-risk enrolled | 3 | 83 | Not reported | 6.5 | 6.63 |
| Montgomery^9^ | Worldwide | Jan-July 21 | Pre-delta (majority alpha) | 46 | Unselected-  90% high-risk | 5 | 0 | 14 | 5.67 | 5.75 |
| Parienti^7^ | France | Nov 20 - March 21 | Pre-delta | 41 | Unselected | 4 | Not reported^#^ | Not reported | 7.22^ | 6.47^ |
| Rossignol^32^ | US, Puerto Rico | Aug 20-Feb 21 | Pre-delta | 40 | Unselected-  62.8% high-risk | 2 | Not reported^#^ | 10 | 6.38 | 6.34 |
| Hammond^27^ | Worldwide | July-Dec 21 | Delta period | 46 | High-risk enrolled | 3 | 0 | 51.2 | 5.41 | 5.3 |
| Fischer^28^ | US | June 20-Jan 21 | Pre-delta | 39 | Unselected-  61% high-risk | 4.5 | 0 | 18.2 | 200mg  5.8  400mg  6.38  800mg  6.69 | 6.11 |
| Weinreich Ph3  1200mg^6^ | US, Mexico | Sep 20- Jan 21 | Pre-delta | 48 | High-risk enrolled | 3 | Not reported^#^ | 23 | 6.73 | 6.63 |
| Weinreich Ph3  2400mg^6^ | US, Mexico | Sep 20- Jan 21 | Pre-delta | 50 | High-risk enrolled | 3 | Not reported^#^ | 23 | 6.72 | 6.66 |
| Dougan  BLAZE-1^29^  Bam-/ etesevimab 2800/2800 | US | Sep-  Dec20 | Pre-delta | 53.8 | High-risk enrolled | 4 | 0 | Not reported | 6.33 | 6.33 |
| Dougan  BLAZE-1^2^  Bam-/etesevimab 700/1400 | US | Dec20-Jan21 | Pre-delta | 56 | High-risk enrolled | 4 | Not  reported^#^ | Not reported | 6.26 | 6.11 |
| Dougan  BLAZE-4^11^ | US | May-July 2021 | Majority delta | 35 | Low-risk enrolled in placebo-controlled cohort | 3 | 0 | 11 | BEB  6.4  BEB+BAM/  ETE  6 | 6 |
| Streinu-Cercel^30^ | Worldwide | Oct-Dec 2020 | Pre-delta | 51 | Unselected –  ~77% high-risk | 3 | 0 | 6.7 | Not reported | Not reported |
| Vega^31^ | Worldwide | Dec 202 - Jan 21 | Pre-delta (majority no VOC) | 43 | Unselected –  28% high-risk | 5 | 43 | 13 | 5mg/kg  5.2  15mg/kg  5.04  30mg/kg  4.81 | 4.7 |
| Bramante  COVID-OUT^33^  Metformin | US | Dec 20-Jan 22 | Majority delta (65%), omicron (22%) | 45.5 | High-risk enrolled | 4.8 | 52 | Not reported | 4.97 | 4.79 |
| Bramante  COVID-OUT^33^  Ivermectin | US | Dec 20-Jan 22 | Majority delta (69%),  omicron (29%) | 45.5 | High-risk enrolled | 4.7 | 56 | Not reported | 4.93 | 4.76 |
| Bramante  COVID-OUT^33^  Fluvoxamine | US | Dec 20-Jan 22 | Majority delta (83%), omicron (13%) | 44.5 | High-risk enrolled | 4.9 | 56 | Not reported | 4.69 | 4.98 |
| Rocco  SARITA-2^34^ | Brazil | June-Aug 20 | Pre-delta | ~39 | Unselected –  15% high-risk | 5 | 0 | Not reported | 7.06 | 7.49 |
| Luvira  PLATCOV^35^ | Thailand, Brazil | Sep 21-Oct 22 | Majority omicron (90%) | 30.1 | Low risk enrolled | 2.2 | 97.5 | Not reported | 5.5 | 5.46 |
| Weinreich Ph2^4^ | US, Mexico | June 20-Aug20 | Pre-delta | 44 | Unselected-  64% | 3 | 0 | 45 | 2400mg  5.04  8000mg  5 | 4.67 |
| Mukae 2b^36^ | Japan, South Korea | Jan-Feb 22 | Majority omicron (99%) | 36 | Unselected | ~3 | 85 | Not reported | 125mg  7.01  250mg  6.82 | 6.88 |
| Khoo^37^ | UK | Nov 20-March 22 | Majority delta (40%) | 43 | Low risk enrolled | 3 | 50 | Not reported | 7.1 | 7.4 |
| Holubar^38^ | US | July 20-March 21 | Pre-delta | 43 | Unselected –  31% high-risk | 5 | 1.7 | 8.6 | 6.41^ | 5.93^ |
| Lowe^39^ | UK | Oct 20-Nov 21 | Pre-delta and delta | 40 | Unselected –  15% high-risk | ~4 | 51 | 63 | Favi+L/R  4.57  Favi  4.23  L/R  4.68 | 4.68 |
| Sirijatuphat^40^ | Thailand | Dec 20-July 21 | Pre-delta (majority alpha) | 30 | Unselected –  ~8% high-risk | 0 | 0 | Not reported | 6.35 | 5.93 |
| Rodrigues ^41^ | Brazil | April-May 20 | Pre-delta | 36.6 | Unselected | ~3.7 | 0 | Not reported | 7.06^ | 6.43^ |
| Buonfrate ^42^ | Italy | July 20- May 21 | Pre-delta | 47 | Unselected-  33% high-risk | 4 | 2.2 | Not reported | 600µg/kg  4.3  1200µg/kg  4.4 | 4.4 |
| De la Rocha^43^ | Mexico | July 20-Jan 21 | Pre-delta | 38.4 | Unselected | Not  reported | Not reported^#^ | Not reported | 5.66^ | 6.53^ |
| Chandiwana^44^ | South Africa | Sep 20- Aug 21 | Pre-delta and delta | 34.9 | Unselected –  ~25% high-risk | 2.7 | <1 | 28.6 | AA  5.57  PA  5.05  FN  5.81  SD  4.91 | 5.79 |
| Schilling  PLATCOV^45^ | Thailand | Sep21-April 22 | Majority delta | 27 | Low risk enrolled | Not reported | Median 2 vaccine doses received | 84 | 5.7 | 5.5 |
| Jittmala  PLATCOV^46^ | Thailand, Brazil | Sep21-June 22 (Remdesivir)  Sep21-Oct 22 (casiri-/imdevimab) | Delta and omicron | 28 | Low risk enrolled | 2 | Median 3 vaccine doses received | 91 | Remdesivir 5.53  Casiri-/imdevimab  5.69 | Remdesivir control  5.63  Casiri-/imdevimab control  5.42 |

*If infecting variants were not specified, likely infecting variants were inferred based on the period of enrolment (pre-delta if enrolment finished prior to July 21)

^Ct values converted to Log10 with formula 45-Ct/Log_2_10 (Ct to Log10 conversion formula used in BLAZE-1 study)

#Assume majority unvaccinated based on enrolment finishing in early 2021

∞Excluded undetectable at all timepoints from individual patient data provided

**Table S4: Baseline characteristics of non-eligible studies**

| **Author** | **Geography** | **Enrolment period** | **Infecting variants*** | **Average age (years)** | **Risk status** | **Average symptom duration (days)** | **% Vaccinated** | **% Sero-positive** | **Baseline VL drug (Log10 copes/ml)** | **Baseline VL control (Log10 copies/ml)** |
| --- | --- | --- | --- | --- | --- | --- | --- | --- | --- | --- |
| Tippabhotla^47^ | India | July – Aug 2021 | Pre-delta/Delta | 36.5 | 7.3% | 45.8% > 3 days | Not  reported | Not  reported | Not  reported | Not  reported |
| Bosaeed^48^ | Saudi Arabia | Jul 2020 – Aug 2021 | Pre-delta/Delta | 37 | Unselected | 3 | Not  reported | Not  reported | Not  reported | Not  reported |
| Golan^49^ | US, Brazil, Mexico | Nov 2020 – Oct 2021 | Pre-delta/Delta | 14.7% ≥60 | 77.8% high risk | 3 | 11% | 29.3% | Not  reported | Not  reported |
| Reis^50^(hydroxychloroquine, lopinavir-ritonavir) | Brazil | Jun 2020 – Sep 2020 | Pre-delta | 53 | High-risk enrolled | 83.8% > 5 days | Not reported^#^ | Not  reported | Not  reported | Not  reported |
| Reis^51^(fluvoxamine) | Brazil | Jan 2020 – Aug 2021 | Pre-delta/Delta | 50 | High-risk enrolled | 3.8 | 6% | Not  reported | Not  reported | Not  reported |
| Reis^52^ (ivermectin) | Brazil | Mar – Aug 2021 | Pre-delta/Delta | 49 | High-risk enrolled | 3.8 | Not  reported | Not  reported | Not  reported | Not  reported |
| Reis^53^(metformin) | Brazil | Jun 2020 – Jan 2021 | Pre-delta | 52 | High-risk enrolled | 35.6% > 3 days | Unvaccinated | Not  reported | Not  reported | Not  reported |
| Reis^54^(fluvoxamine and inhaled budesonide) | Brazil | Jan – July 2022 | Omicron | 51 | High-risk enrolled | 3 | 97.7% | Not  reported | Not  reported | Not  reported |
| Johnston^55^ | US | Apr – Jul 2020 | Pre-delta | 37 | 55.8% high risk | 5.9 | Not  reported^#^ | Not  reported | Not  reported | Not  reported |
| Spivak^56^ | US | Apr 2020 – Apr 2021 | Pre-delta | 41.9 | 24.5% high risk | Not  reported | Not  reported | Not  reported | Not  reported | Not  reported |
| Vallejos^57^ | Argentina | Aug 2020 – Feb 2021 | Pre-delta | 42 | Unselected | 4 | Not  reported | Not  reported | Not  reported | Not  reported |
| Rezai^58^ | Iran | Feb – Aug 2021 | Pre-delta/delta | 35.5 | Unselected | 3.1 | Not  reported | Not  reported | Not  reported | Not  reported |
| Omrani^3^ | Qatar | Apr – Aug 2020 | Pre-delta | 41 | Unselected | Not  reported | Not  reported^#^ | Not  reported | HCQ + azithro = 6.92^  HCQ = 6.51^ | 6.86^ |

*If infecting variants were not specified, likely infecting variants were inferred based on the period of enrolment (pre-delta if enrolment finished prior to July 21, omicron if after November 21)

^Ct values converted to Log10 with formula 45-Ct/Log_2_10 (Ct to Log10 conversion formula used in BLAZE-1 study)

#Assume majority unvaccinated based on enrolment finishing in early 2021

**Table S5: Clinical outcomes for eligible studies**

| **Author** | **Therapy** | **Clinical population** | **Clinical endpoint** | **Risk intervention** | **Risk control** | **Relative risk**  **(95% CI)** |
| --- | --- | --- | --- | --- | --- | --- |
| Chew^12^ | Bamlanivimab 700mg  Bamlanivimab 7000mg | 223  94 | All-cause hosp./death day 28 | 4/111  2/48 | 4/112  4/46 | 1.00 (0.26 – 3.93)  0.48 (0.09 – 2.49) |
| Alemany^13^ | Convalescent plasma | 376 | All-cause hosp./death day 28 | 22/188 | 21/188 | 1.05 (0.60 – 1.84) |
| Gharbaran^14^ | Convalescent plasma | 416 | All-cause hosp./death day 28 | 11/207 | 19/209 | 0.58 (0.29 – 1.20) |
| Evering^15^ | Amubarvimab/romlusevimab | 807 | All-cause hosp./death day 28 | 9/397 | 44/410 | 0.21 (0.10 – 0.43) |
| Bender Ignacio^16^ | Tixa/cilgavimab IM  Tixa/cilgavimab IV | 223  114 | All-cause hosp./death day 28 | 4/106  0/58 | 7/117  4/56 | 0.63 (0.19 – 2.09)  N/A |
| Bernal^17^ | Molnupiravir 800mg | 1408 | All-cause hosp./death day 29 | 48/709 | 68/699 | 0.70 (0.49 – 0.99) |
| Butler^18^ | Molnupiravir 800mg | 25054 | All-cause hosp./death day 28 | 105/12529 | 98/12525 | 1.07 (0.81 – 1.41) |
| Gottlieb^5^ | Remdesivir | 562 | All-cause hosp./death day 28 | 5/279 | 18/283 | 0.28 (0.11-0.74) |
| Gupta^20^ | Sotrovimab | 1057 | All-cause hosp./death day 29 | 6/528 | 30/529 | 0.20 (0.08 – 0.48) |
| Biber^21^ | Ivermectin | 95 | All-cause hosp./death day 14 | 1/50 | 3/45 | 0.3 (0.03 – 2.78) |
| Gottlieb^19^* | Bamlanivimab 700mg  Bamlanivimab 7000mg  Bamlanivimab 2800mg  Bamlanivimab + etesevimab | 257  263  257  268 | COVID-19 related hosp./or ED visit day 29 | 1/101  2/107  2/101  1/112 | 9/156  9/156  9/156  9/156 | 0.17 (0.02 – 1.33)  0.32 (0.07 – 1.47)  0.34 (0.08 – 1.56)  0.15 (0.02 – 1.20) |
| Sinha^22^ | Molnupiravir 800mg | 1218 | All-cause hosp./death day 28 | 9/608 | 26/610 | 0.35 (0.16 – 0.74) |
| Mitja^23^ | Hydroxychloroquine | 293 | All-cause hosp./death day 28 | 8/136 | 11/157 | 0.84 (0.35 – 2.03) |
| Jagannathan^24^ | Interferon lambda-1a | 120 | All-cause hosp./death day 28 | 2/60 | 2/60 | 1 (0.15 – 6.87) |
| McMahon^26^ | Favipiravir | 198 | All-cause hosp./death day 28 | 14/99 | 9/99 | 1.56 (0.71 – 3.43) |
| Kim^25^ | Regdanvimab | 1315 | All-cause hosp. day 28 | 16/656 | 52/659 | 0.31 (0.18 – 0.54) |
| Feld^8^ | Interferon lambda-1a | 60 | All-cause hosp. day 28 | 1/30 | 1/30 | 1 (0.07 – 15.26) |
| Reis^10^ | Interferon lambda-1a | 1919 | COVID-19 related hosp./all cause death day 28 | 24/916 | 40/1003 | 0.66 (0.40 – 1.08) |
| Montgomery^9^* | Tixa-/cilgavimab IM 600mg | 890 | Severe COVID-19 or all cause death | 24/446 | 41/444 | 0.58 (0.36 – 0.95) |
| Parienti^7^ | TDF/FTC | 60 | All-cause hosp./death day 14 | 2/30 | 1/30 | 2.00 (0.19 – 20.90) |
| Rossignol^32^ | nitazoxanide | 379 | All-cause hosp. day 28 | 1/184 | 5/195 | 0.21 (0.03 – 1.80) |
| Hammond^27^* | Nirmatrelvir | 2224 | All-cause serious adverse events. day 34 | 18/1109 | 74/1115 | 0.24 (0.15 – 0.41) |
| Fischer^28^ | Molnupiravir 200mg  Molnupiravir 400mg  Molnupiravir 800mg | 85  124  117 | All-cause hosp./death day 28 | 0/23  2/62  1/55 | 1/62  1/62  1/62 | N/A  2.00 (0.19 – 21.49)  1.13 (0.72 – 17.60) |
| Weinreich^6^ | Casiri-/imdevimab 1200mg  Casiri-/imdevimab 2400mg | 1484  2696 | All-cause hosp./death day 29 | 7/736  20/1355 | 26/748  66/1341 | 0.27 (0.12 – 0.63)  0.30 (0.18 – 0.49) |
| Dougan^29^ | Bam-/etesevimab 2800/2800 | 1035 | COVID-19 related hosp./all cause death day 29 | 11/518 | 36/517 | 0.31 (0.16 – 0.59) |
| Dougan^2^ | Bam-/etesevimab 700/1400 | 769 | COVID-19 related hosp./all cause death day 29 | 4/511 | 15/258 | 0.13 (0.05 – 0.40) |
| Dougan^11^ | Bebtelovimab  Beb-/bam-/etesevimab | 253  255 | COVID-19 related hosp.  day 29 | 2/125  3/127 | 2/128  2/128 | 1.02 (0.15 – 7.16)  1.51 (0.26 – 8.9) |
| Streinu-Cercel^30^ | Regdanvimab 40mg/kg  Regdanvimab 80mg/kg  Regdanvimab combined dose | 204  207  307 | All-cause hosp./death day 28 | 4/100  5/103  9/203 | 9/104  9/104  9/104 | 0.46 (0.15 – 1.45)  0.561 (0.20 – 1.61)  0.512(0.21-1.25) |
| Vega^31^ | BGB-DXP593 5mg/kg  BGB-DXP593 15mg/kg  BGB-DXP593 30mg/kg | 92  90  93 | COVID-19–related hosp. day 113 | 1/45  1/43  0/46 | 2/47  2/47  2/47 | 0.52 (0.05 – 5.56)  0.55 (0.05 – 5.81)  N/A |
| Bramante^33^ | Metformin  Ivermectin  Fluvoxamine | 1307  800  653 | All-cause hosp./death day 14 | 8/652  4/406  6/329 | 18/655  5/394  5/324 | 0.45 (0.20 – 1.02)  0.78 (0.21 – 2.87)  1.18 (0.36 – 3.83) |
| Rocco^34^ | Nitazoxanide | 475 | All-cause hosp. day 14 | 5/238 | 5/237 | 1.00 (0.30 – 3.39) |
| Luvira^35^ | Favipiravir | 248 | All-cause hosp. day 28 | 1/116 | 1/132 | 1.14 (0.07 – 17.97) |
| Weinreich^4^* | Casiri-/imdevimab 2400mg  Casiri-/imdevimab 8000mg | 185  183 | COVID-related medically attended visit day 29 | 3/92  3/90 | 6/93  6/93 | 0.51 (0.13 – 1.96)  0.52 (0.13 – 2.00) |
| Mukae^36^* | Ensitrelvir 125mg  Ensitrelvir 250mg | 281  281 | Serious treatment related adverse event | 0/140  0/140 | 2/141  2/141 | N/A  N/A |
| Khoo^37^ | Molnupiravir 800mg | 180 | All-cause hosp./death day 29 | 0/90 | 4/90 | N/A |
| Holubar^38^ | Favipiravir | 149 | All-cause hosp./death day 28 | 0/75 | 4/74 | N/A |
| Lowe^39^ | Favipiravir,  Lopinavir + ritonavir,  Favipiravir+lopinavir+ritonavir | 121  119  120 | All-cause hosp./death day 28 | 1/61  1/59  1/60 | 0/60  0/60  0/60 | N/A  N/A  N/A |
| Sirijatuphat^40^ | Favipiravir | 93 | All-cause hosp. day 28 | 0/62 | 0/31 | N/A |
| Rodrigues^41^ | Hydroxychloroquine + azithromycin | 84 | All-cause hosp. day 21 | 1/42 | 0/42 | N/A |
| Buonfrate^42^ | Ivermectin 1200 μg/kg  Ivermectin 600 μg/kg | 61  62 | All-cause hosp. day 30 | 1/29  3/30 | 0/32  0/32 | N/A  N/A |
| De la Rocha^43^* | Ivermectin 12mg | 56 | Progression to severe state | 0/30 | 0/26 | N/A |
| Chandiwana^44^ | Artesunate-amodiaquine  Pyronaridine-artesunate  Favipiravir plus nitazoxanide  Sofosbuvir-daclatasvir | 78  75  76  74 | All-cause hosp. day 28 | 0/39  1/36  1/37  1/35 | 0/39  0/39  0/39  0/39 | N/A  N/A  N/A  N/A |
| Schilling^45^ | Ivermectin 600 µg/kg | 91 | All-cause hosp. day 28 | 0/46 | 1/45 | N/A |
| Jittmala^46^ | Remdesivir  Casiri-/imdevimab 1200mg | 136  163 | All-cause hosp. day 28 | 0/67  0/74 | 0/69  1/89 | N/A  N/A |

* Serious adverse events were extracted as a proxy for all-cause hospitalisation or death from the studies of Nirmatrelvir/ritonavir (Hammond *et al*^27^) and Ensitrelvir (Mukae *et al*^36^).

The trial of Tixagevimab/Cilgavimab (Montgomery *et al*^9^) used a primary endpoint of Severe COVID-19 or death from any cause. Since the definition of severe COVID-19 required a WHO clinical progression scale of 5 or more (indicating hospitalisation), this clinical endpoint was considered comparable.

The trial of Ivermectin by De La Rocha *et al*^43^ used a primary endpoint of progression to severe disease; in this trial, patients with early symptomatic COVID-19 were hospitalised for isolation reasons as per national guidelines at the time (i.e. not hospitalised for severe disease). The definition of severe disease in this trial is oxygen saturations <94% on room air or PaO2/FiO2 <300mmHg, respiratory rate >30 breaths/minute, or lung infiltrates >50%. Since these parameters would normally require admission to hospital in outpatients, this endpoint was considered largely comparable to the endpoints of other studies.

The trial of Bamlanivimab by Gottlieb *et al*,^19^ reported COVID-19 related emergency department visits or hospitalisations but did not report hospitalisation only data. Since 12 out of the 15 total ED visits/hospitalisations were hospitalisations, this composite endpoint was considered largely comparable to the endpoints of other studies.

The REGEN phase 2 trial (Weinreich *et al*^4^) combined all COVID-19 related medically attended visits (including telemedicine visits, urgent care, in-person physician visit, ED visit, hospitalisation). This combined endpoint was not considered comparable to hospitalisation or death endpoints and thus was excluded from the meta-regression analysis but the viral load data was used in the secondary analysis which did not require clinical endpoints.

**Table S6: Virologic data for eligible studies**

| **Author** | **Therapy** | **Slope coeff drug** | **Slope coeff**  **control** | **Ratio of slope coefficients** | **Virologic sample size at baseline** | **Data used for slope calculation** | **Sampling days** | **Sampling technique** | **LLOQ** | **LOD** |
| --- | --- | --- | --- | --- | --- | --- | --- | --- | --- | --- |
| Chew^12^ | Bamlanivimab 700mg | -0.545 | -0.516 | 1.06 | 221 | Median viral loads | 0,3,7 | NP | 2Log10 | 1.4Log10 |
| Chew^12^ | Bamlanivimab 7000mg | -0.578 | -0.433 | 1.33 | 93 | Median viral loads | 0,3,7 | NP | 2Log10 | 1.4Log10 |
| Alemany^13^ | Convalescent plasma | -0.467 | -0.468 | 1 | 360 | Median viral loads | 1,3,7 | NP | 2.75Log10 |  |
| Gharbaran^14^ | Convalescent plasma | -0.607 | -0.586 | 1.04 | 84 | Median viral loads* | 1,3,7 | NP |  |  |
| Evering^15^ | Amubarvimab/  Romlusevimab | -0.453 | -0.337 | 1.35 | 735 | Median viral loads | 0,3,7 | Nasal | 2Log10 | 1.4Log10 |
| Bender Ignacio^16^ | IM Tixagevimab/  Cilgavimab | -0.579 | -0.483 | 1.2 | 188 | Median viral loads | 0,3,7 | NP | 2Log10 | 1.4Log10 |
| Bender Ignacio^16^ | IV Tixagevimab/  Cilgavimab | -0.502 | -0.224 | 1.7 | 98 | Median viral loads | 0,3,7 | NP | 2Log10 | 1.4Log10 |
| Bernal^17^ | Molnupiravir 800mg | -0.523 | -0.453 | 1.2 | 1093 | Mean viral loads | 1,3,5 | NP | 500 copies/ml |  |
| Butler^18^ | Molnupiravir 800mg | -0.622 | -0.411 | 1.52 | 73 | Mean viral loads | 1,2,3,4,  5,6,7 | NP/OP |  |  |
| Gottlieb^5^ | Remdesivir | -0.367 | -0.37 | 0.99 | 428 | Mean viral loads | 1,7 | NP | 2228 copies/ml | 1493 copies/ml |
| Gupta^20^ | Sotrovimab | -0.466 | -0.419 | 1.11 | 754 | Median viral loads | 1,5,8 | Nasal secretions | 2228 copies/ml | 1493 copies/ml |
| Biber^21^ | Ivermectin | -0.538 | -0.419 | 1.28 | 89 | Median viral loads* | 0,2,4,6 | NP |  | Ct ≥35 |
| Gottlieb^19^ | Bamlamivimab 700mg | -0.513 | -0.453 | 1.13 | 253 | Mean viral loads | 1,3,7 | NP |  | Ct ≥45 |
| Gottlieb^19^ | Bamlanivimab  2800mg | -0.514 | -0.453 | 1.13 | 259 | Mean viral loads | 1,3,7 | NP |  | Ct ≥45 |
| Gottlieb^19^ | Bamlanivimab  7000mg | -0.5315 | -0.453 | 1.17 | 253 | Mean viral loads | 1,3,7 | NP |  | Ct ≥45 |
| Gottlieb^19^ | Bam-/etesevimab 2800/2800mg | -0.681 | -0.453 | 1.50 | 261 | Mean viral loads | 1,3,7 | NP |  | Ct ≥45 |
| Sinha^22^ | Molnupiravir 800mg | -0.715 | -0.4 | 1.79 | 1218 | Mean viral loads* | 1,5 | NP/OP |  | Ct ≥35 |
| Mitja^23^ | Hydroxychloroquine | -0.569 | -0.531 | 1.07 | 292 | Median viral loads | 1,3,7 | NP | 3Log10 |  |
| Jagannathan^24^ | Interferon Lambda-1a | -0.397 | -0.319 | 1.24 | 106 | Median viral loads | 0,1,3,5,7 | OP |  | Ct ≥42 |
| McMahon^26^ | Favipiravir | -0.92 | -0.805 | 1.14 | 169 | Median viral loads | 1,2,3,4,5,  6,7 | Nasal | 300 copies/ml |  |
| Kim^25^ | Regdanvimab 40mg/kg | -0.49 | -0.368 | 1.33 | 1225 | Mean viral loads | 1,3,7 | NP |  |  |
| Feld^8^ | Interferon Lambda -1a | -0.755 | -0.466 | 1.62 | 60 | Mean viral loads | 0,1,2,3,4,  5,7 | Nasal |  | 20 copies/ml |
| Reis^10^ | Interferon Lambda -1a | -0.795 | -0.682 | 1.17 | 30^ | Mean viral loads | 0,1,2,3,4,  5,6,7 | Nasal |  | 10 copies/µl |
| Montgomery^9^ | IM Tixagevimab/  Cilgavimab | -0.288 | -0.243 | 1.18 | 332 | Mean viral loads | 0,3,6 | Nasal |  |  |
| Parienti^7^ | Tenofovir DF/  emtricitabine | -0.582 | -0.437 | 1.33 | 60 | Unadjusted mean change from baseline* | 1,7 | NP |  | Ct ≥40 |
| Rossignol^32^ | Nitazoxanide | -0.233 | -0.34 | 0.69 | 379 | Unadjusted mean change from baseline | 1,4 | NP |  |  |
| Hammond^27^ | Nirmatrelvir/  Ritonavir | -0.74 | -0.573 | 1.29 | 1761 | Least square mean change from baseline | 1,5 | NP | 2Log10 |  |
| Fischer^28^ | Molnupiravir 200mg | -0.338 | -0.325 | 1.04 | 84 | Least square mean change from baseline | 1,7 | NP | 1018 copies/ml |  |
| Fischer^28^ | Molnupiravir 400mg | -0.377 | -0.325 | 1.16 | 122 | Least square mean change from baseline | 1,7 | NP | 1018 copies/ml |  |
| Fischer^28^ | Molnupiravir 800mg | -0.414 | -0.325 | 1.27 | 114 | Least square mean change from baseline | 1,7 | NP | 1018 copies/ml |  |
| Weinreich Ph3^6^ | Casiri-/imdevimab 1200mg | -0.558 | -0.44 | 1.27 | 1478 | Least square mean change from baseline | 1,7 | NP | 2.85Log10 |  |
| Weinreich Ph3^6^ | Casiri-/imdevimab 2400mg | -0.553 | -0.412 | 1.34 | 2686 | Least square mean change from baseline | 1,7 | NP | 2.85Log10 |  |
| Dougan^29^ | Bam-/etesevimab 2800/2800 | -0.610 | -0.410 | 1.49 | 1035 | Least square mean change from baseline | 1,7 | NP |  | Ct ≥45 |
| Dougan^2^ | Bam-/etesevimab 700/1400 | -0.607 | -0.428 | 1.42 | 769 | Least square mean change from baseline | 1,7 | NP |  | Ct ≥45 |
| Dougan^11^ | Bebtelovimab | -0.628 | -0.603 | 1.04 | 253 | Least square mean change from baseline | 1,7 | NP |  |  |
| Dougan^11^ | Beb-/bam-/etesevimab | -0.667 | -0.603 | 1.11 | 255 | Least square mean change from baseline | 1,7 | NP |  |  |
| Streinu- Cercel^30^ | Regdanvimab 40mg/kg | -0.584 | -0.406 | 1.44 | 204 | Unadjusted mean change from baseline | 1,2,3,4,5,  6,7 | NP |  |  |
| Streinu- Cercel^30^ | Regdanvimab 80mg/kg | -0.577 | -0.406 | 1.42 | 207 | Unadjusted mean change from baseline | 1,2,3,4,5,  6,7 | NP |  |  |
| Streinu- Cercel^30^ | Regdanvimab combined dose | -0.582 | -0.406 | 1.43 | 307 | Unadjusted mean change from baseline | 1,2,3,4,5,  6,7 | NP |  |  |
| Vega^31^ | BGB-DXP59 5mg/kg | -0.481 | -0.446 | 1.08 | 89 | Least square mean change from baseline | 1,8 | NP |  |  |
| Vega^31^ | BGB-DXP59 15mg/kg | -0.519 | -0.446 | 1.16 | 83 | Least square mean change from baseline | 1,8 | NP |  |  |
| Vega^31^ | BGB-DXP59 30mg/kg | -0.418 | -0.446 | 0.94 | 90 | Least square mean change from baseline | 1,8 | NP |  |  |
| Bramante^33^ | Metformin | -1.018 | -0.711 | 1.43 | 945 | Median viral loads | 1,5 | Nasal |  |  |
| Bramante^33^ | Ivermectin | -0.877 | -0.995 | 0.88 | 581 | Median viral loads | 1,5 | Nasal |  |  |
| Bramante^33^ | Fluvoxamine | -0.946 | -0.904 | 1.05 | 471 | Median viral loads | 1,5 | Nasal |  |  |
| Rocco^34^ | Nitazoxanide | -0.686 | -0.672 | 1.02 | 392 | Median viral loads | 0,5 | NP |  |  |
| Luvira^35^ | Favipiravir | -0.377 | -0.404 | 0.93 | 244 | Median viral loads | 0,1,2,3,5,  6,7 | OP |  |  |
| Weinreich Ph2^4^ | Casiri-/imdevimab 2400mg | -0.517 | -0.397 | 1.30 | 154 | Unadjusted mean change from baseline | 1,7 | NP | 2.85Log10 |  |
| Weinreich Ph2^4^ | Casiri-/imdevimab 8000mg | -0.52 | -0.397 | 1.31 | 155 | Unadjusted mean change from baseline | 1,7 | NP | 2.85Log10 |  |
| Mukae 2b^36^ | Ensitrelvir 125mg | -0.830 | -0.482 | 1.72 | 224 | Median viral loads | 1,2,3,4,5,6 | NP | 2.08Log10 |  |
| Mukae 2b^36^ | Ensitrelvir 250mg | -0.738 | -0.482 | 1.53 | 226 | Median viral loads | 1,2,3,4,5,6 | NP | 2.08Log10 |  |
| Khoo^37^ | Molnupiravir | -0.971 | -0.860 | 1.13 | 178 | Mean viral loads | 1,3,5,8 | NP |  | Ct ≥32 |
| Holubar^38^ | Favipiravir | -0.393 | -0.334 | 1.18 | 89 | Mean viral loads | 1,2,3,4,5,  6,7 | Nasal |  | Ct ≥40 |
| Lowe^39^ | Favipiravir plus  lopinavir-ritonavir | -0.484 | -0.421 | 1.15 | 120 | Median viral loads | 1,2,3,4,5,  6,7 | Saliva |  | Ct ≥40 |
| Lowe^39^ | Favipiravir | -0.552 | -0.421 | 1.31 | 119 | Median viral loads | 1,2,3,4,5,  6,7 | Saliva |  | Ct ≥40 |
| Lowe^39^ | Lopinavir-ritonavir | -0.408 | -0.421 | 0.97 | 118 | Median viral loads | 1,2,3,4,5,  6,7 | Saliva |  | Ct ≥40 |
| Sirijatuphat^40^ | Favipiravir | -0.442 | -0.377 | 1.17 | 80 | Median viral loads | 0,1,4,7 | Saliva | 800 copies/ml |  |
| Rodrigues^41^ | Hydroxy-  chloroquine + azithromycin | -0.593 | -0.511 | 1.16 | 84 | Mean viral loads* | 0,3,6 | NP/OP |  | Ct ≥35 |
| Buonfrate^42^ | Ivermectin 600 μg/kg | -0.467 | -0.350 | 1.33 | 57 | Median viral loads | 1,7 | NP |  |  |
| Buonfrate^42^ | Ivermectin 1200 μg/kg | -0.467 | -0.350 | 1.33 | 59 | Median viral loads | 1,7 | NP |  |  |
| De la Rocha^43^ | Ivermectin | -0.335 | -0.373 | 0.90 | 56 | Mean viral loads * | 1,5 | NP |  |  |
| Chandiwana^44^ | Artesunate-amodiaquine | -0.611 | -0.584 | 1.05 | 75 | Median viral loads | 0,3,7 | Nasal |  |  |
| Chandiwana^44^ | Pyronaridine-artesunate | -0.404 | -0.584 | 0.69 | 69 | Median viral loads | 0,3,7 | Nasal |  |  |
| Chandiwana^44^ | Favipiravir plus nitazoxanide | -0.523 | -0.584 | 0.90 | 73 | Median viral loads | 0,3,7 | Nasal |  |  |
| Chandiwana^44^ | Sofosbuvir-daclatasvir | -0.432 | -0.584 | 0.74 | 71 | Median viral loads | 0,3,7 | Nasal |  |  |
| Schilling^45^ | Ivermectin | -0.367 | -0.304 | 1.21 | 93 | Median viral loads | 0,1,2,3,5,  6,7 | OP |  |  |
| Jittmala^46^ | Remdesivir | -0.554 | -0.350 | 1.58 | 134 | Median viral loads | 0,1,2,3,5,  6,7 | OP |  |  |
| Jittmala^46^ | Casiri-/imdevimab 1200mg | -0.499 | -0.382 | 1.31 | 211 | Median viral loads | 0,1,2,3,5,  6,7 | OP |  |  |

*Ct values were converted to Log10 RNA copies/ml using the formula 45-Ct/Log_2_10 (conversion formula used in the BLAZE-1 study).

**Table S7: Meta-regression – 32 studies with at least 1 clinical endpoint in each arm**

| **Label** | **Author/Therapy** | **Relative Risk (95% CI)** | **Ratio of slope coefficients** |
| --- | --- | --- | --- |
| 1 | PANORAMIC Molnupiravir^18^ | 1.07 (0.81 – 1.41) | 1.52 |
| 2 | Fischer Molnupiravir 400mg^28^ | 2.00 (0.19 – 21.49) | 1.16 |
| 3 | Fischer Molnupiravir 800mg^28^ | 1.13 (0.72 – 17.60) | 1.27 |
| 4 | PINETREE Remdesivir^5^ | 0.14 (0.03 – 0.586) | 0.99 |
| 5 | REGEN ph3 1200mg^6^ | 0.27 (0.12 – 0.63) | 1.27 |
| 6 | REGEN Ph3 2400mg^6^ | 0.30 (0.18 – 0.49) | 1.34 |
| 7 | BLAZE-1 ph2 Bam 700mg^19^ | 0.17 (0.02 – 1.33) | 1.13 |
| 8 | BLAZE-1 Ph2 Bam 2800mg^19^ | 0.32 (0.07 – 1.47) | 1.13 |
| 9 | BLAZE-1 ph2 Bam 7000mg^19^ | 0.34 (0.08 – 1.56) | 1.17 |
| 10 | BLAZE-1 ph2 Bam/Ete 2800mg^19^ | 0.15 (0.02 – 1.20) | 1.50 |
| 11 | BLAZE-1 ph3 Bam/Ete 2800mg^29^ | 0.30 (0.16 – 0.60) | 1.49 |
| 12 | BLAZE-1 ph3 Bam/Ete 700/1400mg^2^ | 0.13 (0.05 – 0.40) | 1.42 |
| 13 | Streinu-Cercel ph2  Regdanvimab combined dose ^30^ | 0.512(0.21-1.25) | 1.44 |
| 14 | Kim ph3 Regdanvimab^25^ | 0.31 (0.18 – 0.54) | 1.33 |
| 15 | Mitja Hydroxychloroquine^23^ | 0.84 (0.35 – 2.03) | 1.07 |
| 16 | Alemany Conv. Plasma^13^ | 1.05 (0.60 – 1.84) | 1.00 |
| 17 | Chew Bam- 700mg^12^ | 1.00 (0.25 – 3.96) | 1.06 |
| 18 | Chew Bam- 7000mg^12^ | 0.48 (0.09 – 2.49) | 1.33 |
| 19 | BLAZE-4 Bebtelovimab^11^ | 1.02 (0.15 – 7.16) | 1.04 |
| 20 | BLAZE-4 Beb+Bam/Ete^11^ | 1.51 (0.26 – 9.90) | 1.11 |
| 21 | Feld Interferon Lambda^8^ | 1 (0.07 – 15.26) | 1.62 |
| 22 | TOGETHER Interferon Lambda^10^ | 0.657 (0.40 – 1.08) | 1.17 |
| 23 | Evering Amubarvimab/romlusevimab^15^ | 0.21 (0.10 – 0.43) | 1.35 |
| 24 | Gharbharan Conv.plasma^14^ | 0.58 (0.29 – 1.20) | 1.04 |
| 25 | McMahon Favipiravir^26^ | 1.56 (0.71 – 3.43) | 1.14 |
| 26 | AR0-CORONA TDF/Em^7^ | 2.00 (0.19 – 20.90) | 1.33 |
| 27 | Bender Ignacio IM Tix-/Cil^16^ | 0.63 (0.19 – 2.09) | 1.20 |
| 28 | EPIC HR Nirmatrelvir/ritonavir^27^ | 0.24 (0.15 – 0.41) | 1.29 |
| 29 | MoVE-OUT Molnupiravir^17^ | 0.70 (0.49 – 0.99) | 1.20 |
| 30 | HETERO Molnupiravir^22^ | 0.35 (0.16 – 0.73) | 1.79 |
| 31 | COMET-ICE Sotrovimab^20^ | 0.20 (0.08 – 0.48) | 1.11 |
| 32 | TACKLE IM Tix-/Cil^9^ | 0.50 (0.29 – 0.86) | 1.18 |
| 33 | Vega BGB-DXP59 5mg/kg^31^ | 0.52 (0.05 – 5.56) | 1.08 |
| 34 | Vega BGB-DXP59 15mg/kg^31^ | 0.55 (0.05 – 5.81) | 1.16 |
| 35 | Jagannathan Interferon Lambda^24^ | 1 (0.15 – 6.87) | 1.24 |
| 36 | Rossignol Nitazoxanide^32^ | 0.21 (0.03 – 1.80) | 0.69 |
| 37 | Biber Ivermectin^21^ | 0.3 (0.03 – 2.78) | 1.28 |
| 38 | COVID-OUT Metformin^33^ | 0.45 (0.20 – 1.02) | 1.43 |
| 39 | Rocco Nitazoxanide^34^ | 1.00 (0.30 – 3.39) | 1.02 |
| 40 | PLATCOV Favipiravir^35^ | 1.14 (0.07 – 17.97) | 0.93 |
| 41 | COVID-OUT Ivermectin^33^ | 0.78 (0.21 – 2.87) | 0.88 |
| 42 | COVID-OUT Fluvoxamine^33^ | 1.18 (0.36 – 3.83) | 1.05 |

**Table S8: Interaction analysis including vaccination status and variant status as additional covariates in the model**

When including vaccination status as an interaction term, the slope coefficient represents the change in clinical effect size in the predominantly unvaccinated group compared with the predominantly vaccinated group (reference). When including variant status as an additional covariate in the model, the slope coefficient represents the change in clinical effect size in the pre-delta group compared with the delta/omicron group (reference). The lack of statistical significance associated with either interaction term suggests that the association between viral clearance rate ratio (VCRR) and relative risk of hospitalisation or death, does not depend on either vaccination status or variant status.

| **Additional covariate** | **Categories of each covariate** | **Slope coefficient for each covariate category** | **Slope coefficient for interaction term** |
| --- | --- | --- | --- |
| Interaction between **vaccination status** and VCRR | <50% vaccinated | 1.4 (95% CI -1.3 to 4.1), p=0.3 | -1.6 (95% CI -3.7 to 0.5), p=0.1 |
|  | ≥50% vaccinated (reference category) | -0.5 (95% CI -2.9 to 1.9), p=0.7 |  |
|  | VCRR | 0.2 (95% CI -1.6 to 2), p=0.8 |  |
|  | | | |
| Interaction between **variant status** and VCRR | Pre-delta | 0.2 (95% CI -2.3 to 2.7), p=0.9 | -0.6 (95%CI -2.6 to 1.3), p=0.5 |
|  | Delta and/or Omicron (reference category) | 0.7 (95% CI -1.0 to 2.5), p=0.4 |  |
|  | VCRR | -0.9 (95% CI -2.2 to 0.47), p=0.2 |  |

**References**

1. Rohatgi A. WebPlotDigitizer. 4.6 ed 2022.
2. Dougan M, Azizad M, Mocherla B *et al.* A Randomized, Placebo-Controlled Clinical Trial of Bamlanivimab and Etesevimab Together in High-Risk Ambulatory Patients With COVID-19 and Validation of the Prognostic Value of Persistently High Viral Load. *Clin Infect Dis* 2022; **75**: e440-e9.
3. Omrani AS, Pathan SA, Thomas SA *et al.* Randomized double-blinded placebo-controlled trial of hydroxychloroquine with or without azithromycin for virologic cure of non-severe Covid-19. *EClinicalMedicine*. 2020: **1**; 29.
4. Weinreich DM, Sivapalasingam S, Norton T *et al.* a neutralizing antibody cocktail, in outpatients with Covid-19. *N Engl J Med* 2021 Jan 21; **384**: 238-51.
5. Gottlieb RL, Vaca CE, Paredes R *et al.* Early Remdesivir to Prevent Progression to Severe Covid-19 in Outpatients. *N Engl J Med* 2022; **386**: 305-15.
6. Weinreich DM, Sivapalasingam S, Norton T et al. REGEN-COV Antibody Combination and Outcomes in Outpatients with Covid-19. *N Engl J Med* 2021; **385**: e81.
7. Parienti JJ, Prazuck T, Peyro-Saint-Paul L *et al.* Effect of Tenofovir Disoproxil Fumarate and Emtricitabine on nasopharyngeal SARS-CoV-2 viral load burden amongst outpatients with COVID-19: A pilot, randomized, open-label phase 2 trial. *EClinicalMedicine* 2021; **38**: 100993.
8. Feld JJ, Kandel C, Biondi MJ *et al.* Peginterferon lambda for the treatment of outpatients with COVID-19: a phase 2, placebo-controlled randomised trial. *Lancet Respir Med* 2021; **9**: 498-510.
9. Montgomery H, Hobbs FDR, Padilla F *et al.* Efficacy and safety of intramuscular administration of tixagevimab-cilgavimab for early outpatient treatment of COVID-19 (TACKLE): a phase 3, randomised, double-blind, placebo-controlled trial. *Lancet Respir Med* 2022; **10**: 985-96.
10. Reis G, Moreira Silva EAS, Medeiros Silva DC *et al.* Early Treatment with Pegylated Interferon Lambda for Covid-19. *N Engl J Med* 2023;**388**:518-28.
11. Dougan M, Azizad M, Chen P *et al.* Bebtelovimab, alone or together with bamlanivimab and etesevimab, as a broadly neutralizing monoclonal antibody treatment for mild to moderate, ambulatory COVID-19. *MedRxiv*. **[Preprint].** March 12 2022 [cited 2023 Jan 04]. Available from: https://doi.org/10.1101/2022.03.10.22272100
12. Chew KW, Moser C, Daar ES *et al.* Antiviral and clinical activity of bamlanivimab in a randomized trial of non-hospitalized adults with COVID-19*. Nat Commun*. 2022; **13**: 4931.
13. Alemany A, Millat-Martinez P, Corbacho-Monné M *et al.* High-titre methylene blue-treated convalescent plasma as an early treatment for outpatients with COVID-19: a randomised, placebo-controlled trial. *Lancet Respir Med* 2022; **10**: 278-88.
14. Gharbharan A, Jordans C, Zwaginga L *et al.* Outpatient convalescent plasma therapy for high-risk patients with early COVID-19: a randomized placebo-controlled trial. *Clin Infect Dis* 2023; **29**: 208-14.
15. Evering TH, Chew KW, Giganti MJ *et al.* Safety and Efficacy of Combination SARS-CoV-2 Neutralizing Monoclonal Antibodies Amubarvimab Plus Romlusevimab in Nonhospitalized Patients With COVID-19. *Ann Intern Med* 2023; **176**: 658-666
16. Ignacio RA, Chew KW, Moser C *et al.* Safety and Efficacy of Combined Tixagevimab and Cilgavimab Administered Intramuscularly or Intravenously in Nonhospitalized Patients With COVID-19: 2 Randomized Clinical Trials. *JAMA Netw Open* 2023; **6** :e2310039
17. Jayk Bernal A, Gomes Da Silva MM, Musungaie DB *et al.* Molnupiravir for Oral Treatment of Covid-19 in Nonhospitalized Patients. *N Engl J Med* 2022; **386**: 509-20.
18. Butler CC, Hobbs FDR, Gbinigie OA *et al.* Molnupiravir plus usual care versus usual care alone as early treatment for adults with COVID-19 at increased risk of adverse outcomes (PANORAMIC): an open-label, platform-adaptive randomised controlled trial. *Lancet* 2023; **401**: 281-93.
19. Gottlieb RL, Nirula A, Chen P *et al.* Effect of bamlanivimab as monotherapy or in combination with etesevimab on viral load in patients with mild to moderate COVID-19: a randomized clinical trial. *JAMA*. 2021; **325**: 632-44.
20. Gupta A, Gonzalez-Rojas Y, Juarez E *et al.* Effect of Sotrovimab on Hospitalization or Death Among High-risk Patients With Mild to Moderate COVID-19. *JAMA* 2022; **327**: 1236.
21. Biber A, Harmelin G, Lev D *et al.* The effect of ivermectin on the viral load and culture viability in early treatment of nonhospitalized patients with mild COVID-19–a double-blind, randomized placebo-controlled trial. *Int J Infect Dis* 2022; **122**: 733-40.
22. Sinha S, Kumarasamy N, Suram VK *et al*. Efficacy and Safety of Molnupiravir in Mild COVID-19 Patients in India. *Cureus*. 2022; **14**: e31508.
23. Mitjà O, Corbacho-Monné M, Ubals M *et al.* Hydroxychloroquine for early treatment of adults with mild coronavirus disease 2019: a randomized, controlled trial. *Clin Infect Dis* 2021; **73**: e4073-81.
24. Jagannathan P, Andrews JR, Bonilla H *et al.* Peginterferon Lambda-1a for treatment of outpatients with uncomplicated COVID-19: a randomized placebo-controlled trial. *Nat Commun* 2021; **12**: 1967.
25. Kim JY, Sandulescu O, Preotescu LL *et al*. A Randomized Clinical Trial of Regdanvimab in High-Risk Patients With Mild-to-Moderate Coronavirus Disease 2019. *Open Forum Infect Dis* 2022; **9**: ofac406.
26. McMahon JH, Lau JSY, Coldham A *et al.* Favipiravir in early symptomatic COVID-19, a randomised placebo-controlled trial. *eClinicalMedicine*. 2022; **54**: 101703.
27. Hammond J, Leister-Tebbe H, Gardner A *et al.* Oral Nirmatrelvir for High-Risk, Nonhospitalized Adults with Covid-19. *N Engl J Med* 2022; **386**: 1397-408.
28. Fischer WA, Eron Jr JJ, Holman W *et al.* A phase 2a clinical trial of molnupiravir in patients with COVID-19 shows accelerated SARS-CoV-2 RNA clearance and elimination of infectious virus. *Sci Transl Med* 2021; **14**: eabl7430.
29. Dougan M, Nirula A, Azizad M *et al.* Bamlanivimab plus etesevimab in mild or moderate Covid-19. *NEJM* 2021; **385**: 1382-92
30. Streinu-Cercel A, Săndulescu O, Preotescu LL *et al.* Efficacy and safety of regdanvimab (CT-P59): a phase 2/3 randomized, double-blind, placebo-controlled trial in outpatients with mild-to-moderate coronavirus disease 2019. *Open Forum Infect Dis* 2022; **9:** ofac053
31. Vega R, Antila M, Perez C *et al.* SARS-CoV-2-neutralising antibody BGB-DXP593 in mild-to-moderate COVID-19: a multicentre, randomised, double-blind, phase 2 trial. *EClinicalMedicine*. 2023; **57:** 101832
32. Rossignol JF, Bardin MC, Fulgencio J *et al.* A randomized double-blind placebo-controlled clinical trial of nitazoxanide for treatment of mild or moderate COVID-19. EClinicalMedicine. 2022; **45**: 101310
33. Bramante CT, Huling JD, Tignanelli CJ *et al*. Randomized trial of metformin, ivermectin, and fluvoxamine for Covid-19. *N Engl J Med* 2022; **387**: 599-610.
34. Rocco PR, Silva PL, Cruz FF *et al.*  Early use of nitazoxanide in mild Covid-19 disease: randomised, placebo-controlled trial. *Eur Respir J* 2021; **58**: 2003725
35. Luvira V, Schilling WH, Jittamala P *et al.* Clinical antiviral efficacy of favipiravir in early COVID-19 (PLATCOV): an open-label, randomised, controlled, adaptive platform trial. *BMC Infect. Dis*. 2024; **24**: 89.
36. Mukae H, Yotsuyanagi H, Ohmagari N *et al*. Efficacy and Safety of Ensitrelvir in Patients With Mild-to-Moderate Coronavirus Disease 2019: The Phase 2b Part of a Randomized, Placebo-Controlled, Phase 2/3 Study. *Clin Infect Dis* 2023; **76**: 1403-11.
37. Khoo SH, FitzGerald R, Saunders G *et al.* Molnupiravir versus placebo in unvaccinated and vaccinated patients with early SARS-CoV-2 infection in the UK (AGILE CST-2): a randomised, placebo-controlled, double-blind, phase 2 trial. *Lancet Infect Dis* 2023; **23**: 183-95.
38. Holubar M, Subramanian A, Purington N *et al.* Favipiravir for treatment of outpatients with asymptomatic or uncomplicated coronavirus disease 2019: a double-blind, randomized, placebo-controlled, phase 2 trial*. Clin Infect Dis* 2022; **75**: 1883-92.
39. Lowe DM, Brown LA, Chowdhury K *et al.*  Favipiravir, lopinavir-ritonavir, or combination therapy (FLARE): A randomised, double-blind, 2× 2 factorial placebo-controlled trial of early antiviral therapy in COVID-19. *PLoS Med*. 2022; **19**: e1004120.
40. Sirijatuphat R, Manosuthi W, Niyomnaitham S *et al.* Early treatment of Favipiravir in COVID-19 patients without pneumonia: a multicentre, open-labelled, randomized control study*. Emerg microbes & infect* 2022; **11**: 2197-206.
41. Rodrigues C, Freitas-Santos RS, Levi JE *et al.* Hydroxychloroquine plus azithromycin early treatment of mild COVID-19 in an outpatient setting: a randomized, double-blinded, placebo-controlled clinical trial evaluating viral clearance. *Int J Antimicrob Agents* 2021; **58**: 106428.
42. Buonfrate D, Chesini F, Martini D *et al.* High-dose ivermectin for early treatment of COVID-19 (COVER study): a randomised, double-blind, multicentre, phase II, dose-finding, proof-of-concept clinical trial. *Int. J. Antimicrob*. Agents. 2022; **59**: 106516.
43. de la Rocha C, Cid-López MA, Venegas-López BI *et al.* Ivermectin compared with placebo in the clinical course in Mexican patients with asymptomatic and mild COVID-19: a randomized clinical trial. *BMC Infect Dis* 2022; **22**: 917.
44. Chandiwana N, Kruger C, Johnstone H *et al*. Safety and efficacy of four drug regimens versus standard-of-care for the treatment of symptomatic outpatients with COVID-19: A randomised, open-label, multi-arm, phase 2 clinical trial. *eBioMedicine* 2022; **86**: 104322.
45. Schilling WH, Jittamala P, Watson JA *et al*. Pharmacometric assessment of the in vivo antiviral activity of ivermectin in early symptomatic COVID-19. *eLife* 2023; **12**: e83201
46. Jittamala P, Schilling WH, Watson JA *et al.* Clinical antiviral efficacy of remdesivir and casirivimab/imdevimab against the SARS-CoV-2 Delta and Omicron variants. *J Infect Dis.* 2023; **228**: 1318-1325.
47. Tippabhotla SK, Lahiri DS, Kandi C. Efficacy and safety of molnupiravir for the treatment of non-hospitalized adults with mild COVID-19: a randomized, open-label, parallel-group phase 3 trial. The Lancet Preprints **[Preprint]** February 24, 2022 [cited 2024 Jan 4]. Available from https://dx.doi.org/10.2139/ssrn.4042673
48. Bosaeed M, Alharbi A, Mahmoud ­*et al.*  Efficacy of favipiravir in adults with mild COVID-19: a randomized, double-blind, multicentre, placebo-controlled clinical trial. *Clin Microbiol Infect* 2022; **28**: 602-8.
49. Golan Y, Campos JA, Woolson R *et al.* Favipiravir in Patients With Early Mild-to-moderate Coronavirus Disease 2019 (COVID-19): A Randomized Controlled Trial. *Clin Infect Dis* 2023; **76**: e10-7.
50. Reis G, Silva EA, Silva DC *et al.* Effect of early treatment with hydroxychloroquine or lopinavir and ritonavir on risk of hospitalization among patients with COVID-19: the TOGETHER randomized clinical trial*. JAMA Netw Open* 2021; **4**: e216468-.
51. Reis G, dos Santos Moreira Silva EA, Medeiros Silva DC *et al.* Oral Fluvoxamine With Inhaled Budesonide for Treatment of Early-Onset COVID-19: A Randomized Platform Trial. *Ann Intern Med* 2023; **176**: 667-75.
52. Reis G, Silva EA, Silva DC *et al.* Effect of early treatment with ivermectin among patients with Covid-19. *N Engl J Med* 2022; **386**: 1721-31.
53. Reis G, Silva EA, Silva DC *et al.* Effect of early treatment with metformin on risk of emergency care and hospitalization among patients with COVID-19: The TOGETHER randomized platform clinical trial. *Lancet Reg Health Am*. 2022; **6**: 100142
54. Reis G, dos Santos Moreira Silva EA, Medeiros Silva DC *et al.* Oral Fluvoxamine With Inhaled Budesonide for Treatment of Early-Onset COVID-19: A Randomized Platform Trial. *Ann Intern Med* 2023; **176**: 667-75.
55. Johnston C, Brown ER, Stewart J *et al.*  Hydroxychloroquine with or without azithromycin for treatment of early SARS-CoV-2 infection among high-risk outpatient adults: a randomized clinical trial. *EClinicalMedicine*. 2021; **33**: 100773
56. Spivak AM, Barney BJ, Greene T, *et al.* A Randomized Clinical Trial Testing Hydroxychloroquine for Reduction of SARS-CoV-2 Viral Shedding and Hospitalization in Early Outpatient COVID-19 Infection. *Microbiol Spectr* 2023; **13**: e04674-22.
57. Vallejos J, Zoni R, Bangher M, Ivermectin to prevent hospitalizations in patients with COVID-19 (IVERCOR-COVID19) a randomized, double-blind, placebo-controlled trial. *BMC Infect Dis* 2021; **21**: 635
58. Rezai MS, Ahangarkani F, Hill A *et al.* Non-effectiveness of ivermectin on inpatients and outpatients with COVID-19; results of two randomized, double-blinded, placebo-controlled clinical trials*. Front Med* 2022; **16**: 919708.
